# Advanced in vitro cardiac models for drug evaluation: integration of organoids, engineered tissues, and microphysiological systems

**DOI:** 10.1038/s41378-026-01249-6

**Published:** 2026-05-07

**Authors:** Young Hyun Kim, Young Hoon Son, Yuri Choi, Min Suk Kim, Sung-Jin Park, Keel Yong Lee

**Affiliations:** 1 Department of Integrative Bioscience and Biotechnology, Sejong University, Seoul, Republic of Korea; 2 Wallace H. Coulter Department of Biomedical Engineering, Georgia Institute of Technology and Emory University, Atlanta, GA USA; 3 Institute of Bioscience and Biotechnology, Sejong University, Seoul, Republic of Korea

**Keywords:** Biosensors, Environmental, health and safety issues

## Abstract

In vitro cardiac model systems have rapidly advanced as complementary platforms to conventional two-dimensional (2D) cultures and animal models, which, despite their long-standing contributions, exhibit inherent limitations in predicting human cardiac responses. This review highlights recent progress in biomimetic platforms that more faithfully recapitulate the structure and function of the human myocardium, including engineered three-dimensional (3D) tissues, chambered ventricular constructs, self-organizing cardiac organoids, and microphysiological systems. These models are increasingly being applied as Drug Development Tools (DDTs) for safety pharmacology, efficacy testing, and cardiotoxicity assessment, offering improved predictive performance compared to traditional assays. By incorporating key features, such as three-dimensional tissue architecture, multicellular composition, electromechanical coupling, and physiological loading, these platforms enhance the translational relevance of preclinical studies. Recent innovations include maturation-enhanced organoids, vascularized engineered heart tissues, chamber models with physiological pressure–volume dynamics, and chip-based platforms that enable the real-time assessment of contractility and electrophysiology. Importantly, the integration of immune and vascular components, as well as multi-organ connectivity, further extends their applicability to systemic drug evaluations and disease modeling. Collectively, these advances bridge the gap between reductionist in vitro assays and clinical studies and align with emerging regulatory paradigms that emphasize human-relevant and non-animal testing methods. By enabling mechanistic insights into human cardiogenesis, cardiomyocyte maturation, and patient-specific disease modeling, advanced in vitro cardiac platforms hold great promise for precision pharmacology and regenerative medicine. Overall, in vitro cardiac models represent a transformative paradigm for advancing drug discovery, improving safety predictions, and reducing the reliance on animal testing in cardiovascular research.

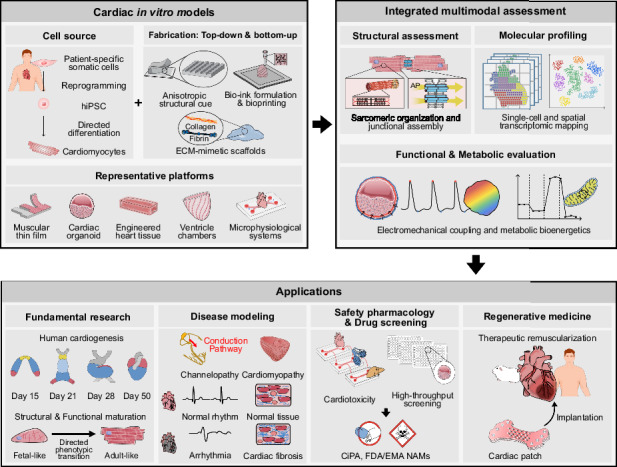

## Introduction

Cardiovascular disease (CVD) is the leading cause of mortality worldwide, accounting for approximately one-third of all global deaths^1^. Despite this substantial disease burden, the development of new cardiovascular therapeutics continues to face high attrition rates during Phase II and III clinical trials, largely due to insufficient efficacy and unexpected toxicities^2,3^. A fundamental limitation underlying these failures is the poor predictive accuracy of conventional preclinical models, which fail to faithfully recapitulate key aspects of human cardiac physiology and pharmacodynamics^4^. Although animal models have long served as the backbone of preclinical cardiovascular research, they differ markedly from humans in heart size, ion channel expression profiles, metabolic pathways, and drug responses. These differences result in significant interspecies variability and limit translational predictability^4,5^. Similarly, widely used in vitro assays, such as hERG ion channel testing in transfected cell lines, capture only isolated electrophysiological effects. As a result, these assays often generate false-positive or false-negative predictions of arrhythmia risk^6,7^. Consequently, clinically relevant cardiac safety liabilities, including proarrhythmic and cardiodepressive effects, may remain undetected until late-stage development or even after market approval^8^. Therefore, drug-induced cardiotoxicity remains a major cause of late-stage clinical trial failure and post-marketing withdrawal, underscoring the urgent need for more predictive, human-relevant cardiac screening platforms. Human induced pluripotent stem cell–derived cardiomyocytes (hiPSC-CMs) have emerged as a promising solution to bridge this translational gap^9^. Reprogrammed from adult somatic cells, hiPSC-CMs retain the human genetic background and recapitulate the key electrophysiological properties of native cardiomyocytes^10,11^. Importantly, they can be generated at scale, maintained in long-term cultures, and integrated into reproducible, high-throughput drug screening workflows^12^. Their intrinsic human relevance enables the detection of cardiotoxic and arrhythmogenic effects that are frequently overlooked in animal models^13^, representing a critical advantage over conventional approaches. Furthermore, patient-specific and gene-edited hiPSC-CMs enable the modeling of a broad spectrum of cardiovascular diseases, ranging from inherited channelopathies to complex cardiomyopathies, thereby advancing precision pharmacology^9^. Contemporary in vitro cardiac models now span a wide continuum of structural and functional complexity. These range from simple two-dimensional (2D) monolayers to three-dimensional (3D) engineered heart tissues (EHTs), self-organizing cardiac organoids (COs), and microphysiological systems (MPSs), commonly referred to as heart-on-a-chip (HoC) platforms^10,14^. Each model class offers a distinct balance between experimental throughput and physiological fidelity, providing complementary tools for efficacy testing, safety, and mechanistic interrogation. In this review, we summarize the current landscape of hiPSC-derived cardiac model systems, critically evaluate their applications in preclinical drug development, and discuss persistent challenges, including tissue maturation, scalability, reproducibility, and regulatory validation of these models. Particular emphasis is placed on recent micro- and nanoscale engineering innovations that are driving these platforms toward enhanced translational relevance and clinical impacts.

## In vitro cardiac model platforms for drug evaluation

**Fig. 1:**
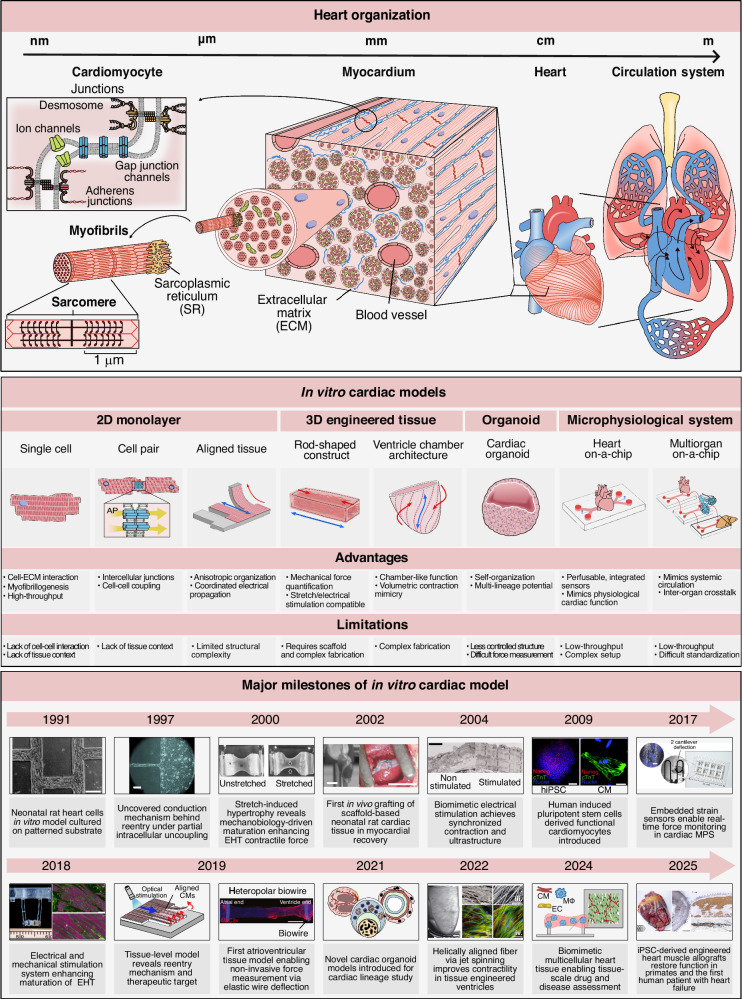
Multiscale overview of in vitro cardiac models, spanning single-cell systems to multi-organ platforms, and illustrating structural hierarchy (top), representative model platforms and their characteristics (middle), and major developmental and technological milestones in the field (bottom). The top panel depicts cardiac organization across length scales, from subcellular structures and cardiomyocytes to myocardium, whole heart, and the circulation system. The middle panel summarizes major classes of in vitro cardiac models, including 2D monolayers, 3D engineered tissues, cardiac organoids, and microphysiological systems, highlighting their representative architectures, advantages, and limitations. The bottom timeline highlights key milestones in the evolution of in vitro cardiac models and is reproduced with permission from the indicated references (1991–2025); scale bars are shown for each representative image: (scale bar = 200 µm) ^191^(1991), (scale bar = 100 µm) ^192^(1997), ^193^(2000), (scale bar = 1 cm) ^194^(2002), (scale bar = 2 µm) ^195^(2004), [scale bars = 100 µm (left), 20 µm (right)] ^196^(2009), ^20^(2017), [scale bars = 100 µm (right-top), 20 µm (right-bottom)]^29^ (2018), (scale bar = 0.5 mm)^30^ (2019), (2021), [scale bars = 2 mm (left), 50 µm (right)]^42^ (2022), (2024), [scale bars = 2 mm (left), 1 cm (middle), 2 mm (right-top), 200 µm (right-bottom)]^33^ (2025). Red arrows indicate contraction direction; blue arrows, relaxation direction. SR sarcoplasmic reticulum, ECM extracellular matrix, AP action potential, EHT engineered heart tissue, hiPSC human induced pluripotent stem cells, CM cardiomyocyte, MPS microphysiological system, MΦ macrophage, EC endothelial cell

To overcome the inherent limitations of conventional in vivo animal models and overly reductionist in vitro assays, a wide range of in vitro cardiac platforms has been developed to improve the predictive accuracy of preclinical cardiovascular drug evaluation (Fig. 1). Whereas early cardiac models relied predominantly on animal-derived cells, such as neonatal rat ventricular myocytes (NRVMs), the field has rapidly transitioned toward human-based systems derived from human induced pluripotent stem cells (hiPSCs) to recapitulate human-specific cardiac physiology more closely. Collectively, these platforms span multiple levels of cardiac organization, ranging from single cardiomyocytes to multicellular engineered tissues and organ-level structures. Each model class entails a distinct trade-off between physiological fidelity and experimental throughput, enabling investigators to select the most appropriate platform based on their specific pharmacological objectives.

In this section, we systematically categorize and evaluate the major classes of in vitro cardiac models, including single-cell assays, geometrically patterned monolayers, three-dimensional engineered heart tissues, cardiac organoids, and microphysiological systems (MPSs). Particular emphasis is placed on their applications in drug efficacy testing, toxicity screening, and cardiac safety pharmacology.

### Single-cell, cell-pair, and aligned monolayer in vitro models

**Fig. 2:**
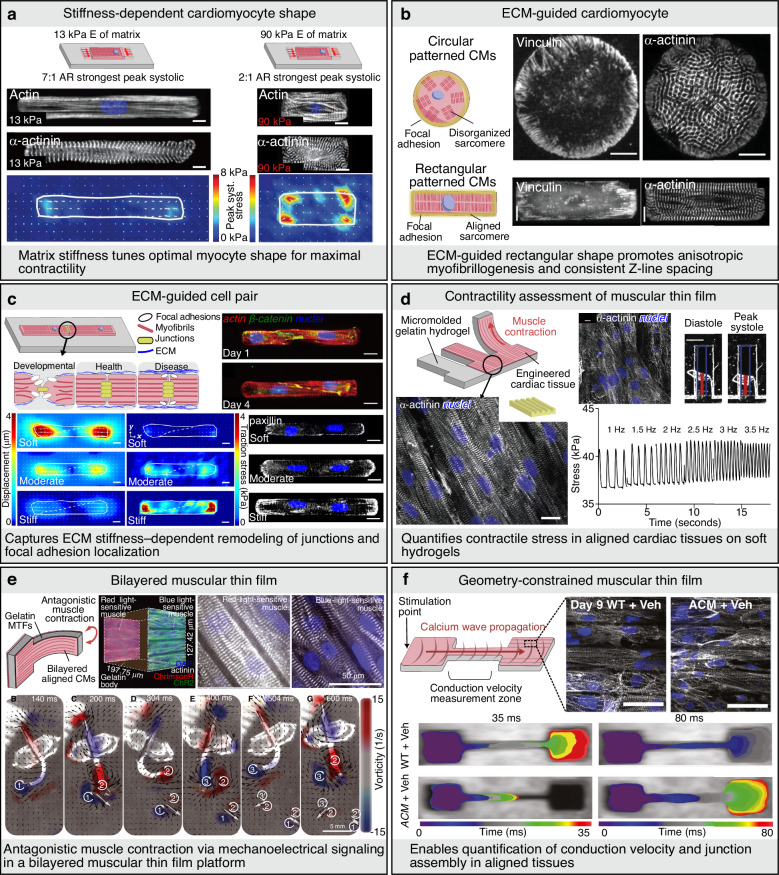
Single cell, cell pair, and aligned monolayer models. **a** Actin alignment is regulated by myocyte shape but not matrix elasticity, sarcomere content is dependent on myocyte shape and matrix elasticity (scale bars = 10 µm). Reproduced with permission^16^. Copyright 2014, The American Physiological Society. **b** CMs on circular and rectangular microcontact printed ECM island (scale bars = 10 µm). Reproduced with permission^15^. Copyright 2008, John Wiley and Sons. **c** (Top) cell–cell junctions had sigmoid-like contours. (Bottom) regulation of displacement, traction stress, and focal adhesion formation by substrate stiffness (scale bars = 10 µm). Reproduced with permission^17^. Copyright 2012, The National Academy of Sciences. **d** (Left) Engineering anisotropic cardiac tissues with micro-molded gelatin hydrogels. (Right) Extended culture of human iPSC-derived cardiac myocytes on micro-molded gelatin MTFs [scale bars = 10 µm (top-left), 1 mm (top-right), 10 µm (bottom)]. Reproduced with permission^18^. Copyright 2014, Elsevier. **e** Muscular bilayer construct showing representative mesoarchitecture and microarchitecture. Body kinematics and hydrodynamics of the biohybrid fish during one and a half tail-beat cycles [scale bars = 50 µm (top), 5 mm (bottom)]. Reproduced with permission^22^. Copyright 2022, The American Association for the Advancement of Science. **f** Calcium wavefront isochrone maps (scale bars = 50 µm). Reproduced with permission^23^. Copyright 2023, Elsevier. ACM arrhythmogenic cardiomyopathy, AR aspect ratio, ChR2 channelrhodopsin-2, CM cardiomyocyte, E elastic modulus, ECM extracellular matrix, MTF muscular thin film, Veh vehicle, WT wild type

The increasing availability of human-induced pluripotent stem cell–derived cardiomyocytes (hiPSC-CMs) has substantially accelerated the development of reproducible and human-relevant in vitro cardiac models^4^. Among the foundational enabling technologies, microcontact patterning provides precise control over cell–extracellular matrix (ECM) interactions and cellular geometries. Early studies demonstrated that single cardiomyocytes cultured on rectangular micropatterns with a 7:1 aspect ratio preferentially formed focal adhesions at the corners, thereby promoting anisotropic actin polymerization and aligned myofibrillar assembly^15^ (Fig. 2a). Subsequent comparisons between circular and rectangular geometries further confirmed that anisotropic constraints enhanced sarcomere organization^16^ (Fig. 2b). Extending these approaches to cell-pair configurations enabled a detailed investigation of electromechanical coupling and junctional protein dynamics under defined substrate stiffness conditions^17^ (Fig. 2c). Building on this concept, anisotropic monolayer systems, often referred to as “2.5D tissues,” have been developed. In these platforms, cardiomyocytes align on linearly patterned substrates, enabling coordinated contraction, junctional maturation, and directionally constrained action potential propagation. The mechanical performance of these aligned monolayers was quantitatively evaluated using muscular thin-film (MTF) platforms, initially fabricated on polydimethylsiloxane (PDMS) substrates to measure contractile bending forces, and later adapted with gelatin-based materials to improve physiological stiffness and long-term culture stability^18^ (Fig. 2d). Subsequent innovations, including optogenetic pacing–enabled MTFs (Opto-MTFs), provided spatiotemporal control of contraction while enabling simultaneous calcium imaging^19^. In parallel, multimaterial three-dimensional printing strategies have facilitated the integration of embedded strain sensors for high-content pharmacological analysis^20^. Additional refinements, such as fibronectin-based micropatterns^21^ and geometric bilayer constructs incorporating insulated pacemaker nodes^22^ (Fig. 2e), further advanced studies on wavefront initiation and propagation dynamics. Narrowed MTF geometries subsequently improved the precision of conduction velocity measurements, enhancing their utility for arrhythmia modeling and drug screening^23^ (Fig. 2f).

Beyond geometric and structural control, hiPSC-CM monolayers constitute a robust and scalable platform for early-stage in vitro drug testing of human cardiac tissue. When cultured as confluent, electrically coupled monolayers in multiwell plate formats, these systems are highly amenable to high-throughput screening using automated imaging, electrophysiological, and impedance-based analyses. Within the Comprehensive in vitro Proarrhythmia Assay (CiPA) framework, 2D hiPSC-CM monolayers serve as the primary human cell–based model. These systems enable the evaluation of integrated electrophysiological responses and improve the prediction of proarrhythmic risk beyond conventional hERG assays^24^. Although these two-dimensional systems cannot fully recapitulate the biomechanical complexity or three-dimensional architecture of the native myocardium, their high reproducibility, scalability, and compatibility with industrial screening workflows render them indispensable for initial cardiotoxicity assessment^25^. Accordingly, hiPSC-CM monolayers provide a critical foundation for preclinical cardiac pharmacology and serve as a practical entry point for more physiologically sophisticated three-dimensional engineered heart tissues and chambered cardiac constructs.

### Advanced 3D models of engineered heart tissue and ventricular architecture

Three-dimensional (3D) in vitro cardiac models provide advanced platforms that more closely recapitulate human cardiac development and physiology than conventional two-dimensional (2D) cultures do. Although 2D monolayers are widely employed for high-throughput applications, three-dimensional engineered tissues offer substantially enhanced structural and functional fidelity, enabling the detection of pathophysiological phenotypes that are often undetectable in planar systems. For example, titin (*TTN*) mutation–associated contractile dysfunction in hiPSC-CMs has been revealed exclusively in three-dimensional microtissue models^26^. Based on their architectural and functional design principles, advanced 3D cardiac models are broadly classified into two major categories: engineered heart tissue (EHTs) and ventricle-like chamber constructs. Each class offers distinct advantages for modeling cardiac mechanics, electrophysiological behavior, and disease-specific phenotypes in humans.

#### Engineered heart tissues (EHTs)

**Fig. 3:**
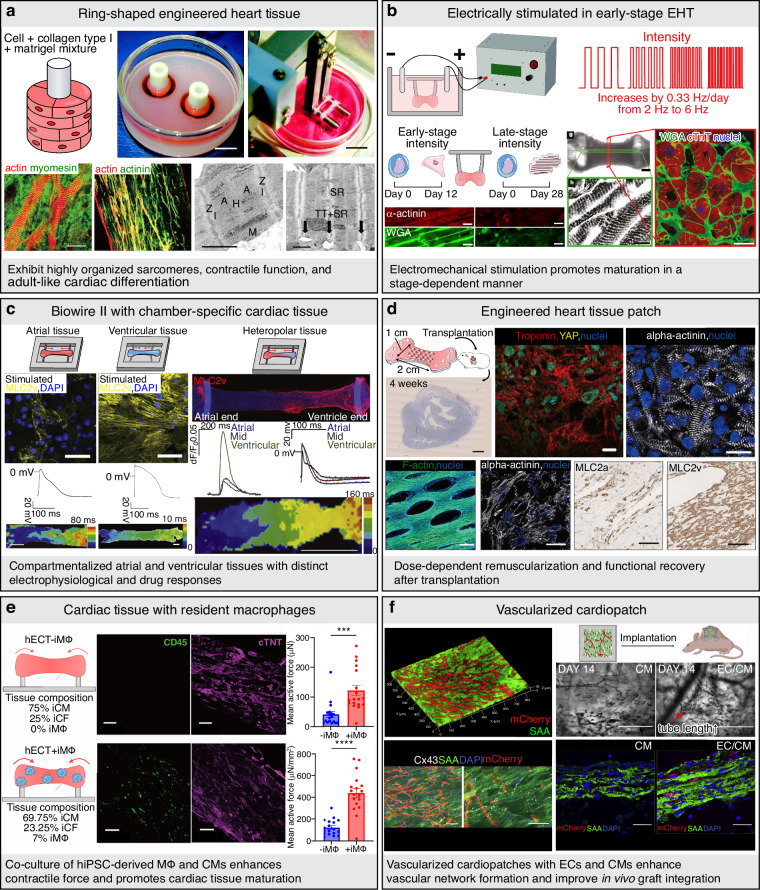
Engineered heart tissue models. **a** (Top) EHT condensation around the central Teflon cylinder in casting molds between culture days 1 to 4. EHTs after transfer in a stretch apparatus to continue culture under unidirectional and cyclic stretch (10%, 2 Hz) [scale bars = 10 mm (top), 20 µm (bottom-1), 100 µm (bottom-2), 1 µm (bottom-3, 4)]. Reproduced with permission^28^. Copyright 2002, Wolters Kluwer Health, Inc. (Bottom) EHTs consist of a dense network of mainly longitudinally oriented cell bundles. TEM of sarcomeric structures, cell–cell junctions, and basal membrane of cardiac myocytes in EHT. **b** Experimental design: early-stage or late-stage hiPSC-CMs and supporting fibroblasts were encapsulated in fibrin hydrogel to form tissues stretched between two elastic pillars and made to contract by electrical stimulation. Cross-sections taken to evaluate T-tubules [scale bars = 20 µm (left-1, 2, 3, 4), 500 µm (middle-top), 10 µm (middle-bottom), 10 µm (right)]. Reproduced with permission^29^. Copyright 2018, Springer Nature. **c** Biowire II platform generating atrial and ventricular-specific, and heteropolar cardiac tissues [scale bars = 30 µm (left-top-1, 2), 0.5 mm (left-bottom-1), 200 µm (left-bottom-2), 2 mm (right)]. Reproduced with permission^30^. Copyright 2019, Elsevier. **d** Large-scale EHT patches restoring heart function in a dose-dependent manner [scale bars = 2 mm (top-left), 20 µm (top-middle), 20 µm (top-right), 500 µm (bottom-1), 50 µm (bottom-2), 250 µm (bottom-3, 4)]. Reproduced with permission^31^. Copyright 2021, Wolters Kluwer Health, Inc. **e** Human engineered cardiac tissue (hECT) integrated with cardiac macrophages to enhance contractility (scale bars = 100 µm). Reproduced with permission^34^. Copyright 2024, Elsevier. **f** Pre-vascularized cardiac patches improving in vivo perfusion and function [scale bars = 1 mm (top), 100 µm (bottom-1), 25 µm (bottom-2, 3, 4)]. Adapted with permission^35^. Copyright 2024, Elsevier. A A-band, CD45 Leukocyte common antigen, CM Cardiomyocyte, cTnT Cardiac troponin T, Cx43 Connexin 43, DAPI 4′,6-diamidino-2-phenylindole, EC endothelial cell, H H-zone, hECT human engineered cardiac tissue, I I-band, iCF induced cardiac fibroblast from hiPSC, iCM induced cardiomyocyte from hiPSC, iMΦ induced macrophage from hiPSC, M M-line, mCherry fluorescent protein (red marker), MLC2a myosin light chain 2a, MLC2v myosin light chain 2v, SAA serum amyloid A, SR sarcoplasmic reticulum, TT T-tubule, WGA wheat germ agglutinin, YAP yes-associated protein, Z Z-line, α-actinin alpha-actinin

EHTs aim to recapitulate the myocardial microenvironment by integrating hiPSC-CMs with ECM components and applying defined mechanical and/or electrical stimulation^27^. Early ring-shaped EHT constructs enabled the quantitative measurement of contractile force within looped geometries while promoting sarcomeric alignment^28^ (Fig. 3a). Subsequent studies have demonstrated that electromechanical stimulation initiated at early developmental stages robustly enhances EHT maturation, leading to adult-like ultrastructural features, organized sarcomeres, T-tubule formation, and more physiological calcium handling^29^ (Fig. 3b). The Biowire II platform further advances EHT technology by generating atrial- and ventricular-specific tissues through spatially confined electrical conditioning, thereby enabling chamber-specific drug response testing^30^ (Fig. 3c). Recent efforts have focused on improving tissue maturity, multicellular complexity, and physiological relevance. In this context, EHT patches and multilayered human cardiac muscle patches (hCMPs) have demonstrated enhanced contractile performance and remuscularization in vivo, achieving functional repair and electromechanical integration in both primate and human hearts. These results represent a major translational milestone for EHT-based cardiac therapies^31,32^ (Fig. 3d). In parallel, large-animal studies have reported that epicardial EHT allografts support long-term engraftment, vascular integration, and recovery of ventricular function in rhesus macaques without inducing arrhythmias or tumorigenesis, further reinforcing clinical feasibility and highlighting critical translational considerations, including graft maturity, immune modulation, and Good Manufacturing Practice (GMP)-scale production^33^. Beyond cardiomyocyte-only constructs, the incorporation of non-myocyte populations, such as cardiac macrophages, has been shown to enhance hiPSC-CM alignment and force generation^34^ (Fig. 3e). In addition, prevascularization strategies employing endothelial cells (ECs) and fibroblasts (FBs) have further improved tissue integration, perfusion, and functional stability after transplantation^35^ (Fig. 3f).

#### Ventricle chamber models

**Fig. 4:**
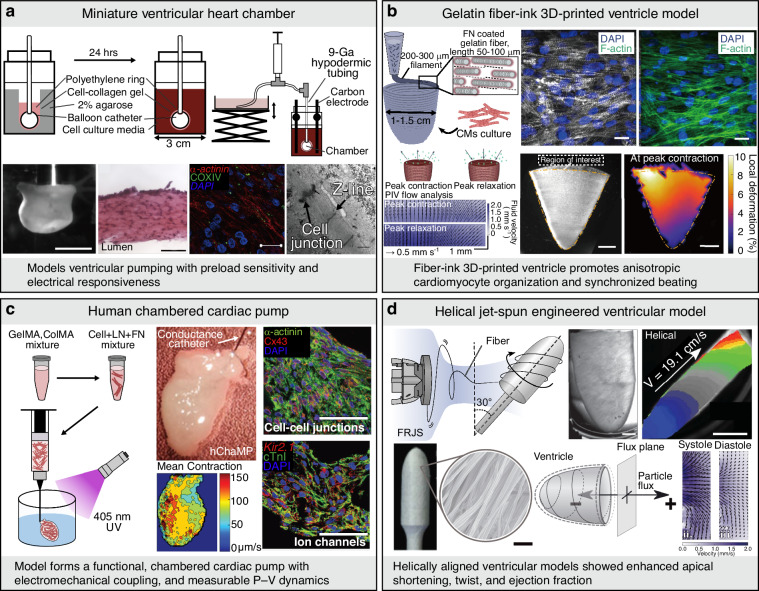
Ventricle chamber models. **a** Engineering human ventricular cardiac organoid chambers (hvCOC) [scale bars = 5 mm (1), 50 µm (2), 20 µm (3), 500 nm (4)]. Reproduced with permission^37^. Copyright 2018, Elsevier. **b** Fiber alignment that occurs under shear stress (τ) during 3D printing leads to native ECM anisotropic structural features in 3D scaffolds, promoting tissue alignment and organization to recapitulate in vivo heart muscle. Structural, electrophysiological, and contractile properties of 3D ventricle models (scale bar = 50 µm). Reproduced with permission^40^. Copyright 2020, Wolters Kluwer Health, Inc. **c** The optimized bioink formulation was combined with human iPSCs and bioprinted to form a hChaMP [scale bars = 2 mm (top-left), 5 mm (top-right), 5 mm (bottom-left), 5 µm (bottom-right)]. Reproduced with permission^41^. Copyright 2022, The American Association for the Advancement of Science. **d** Focused rotary jet spinning for producing helical structures [scale bars = 20 µm (top), 2 mm (bottom)]. Reproduced with permission^42^. Copyright 2023, The American Association for the Advancement of Science. COXIV cytochrome c oxidase subunit IV, CMs cardiomyocytes, cTnI cardiac troponin I, Cx43 Connexin 43, DAPI 4′,6-diamidino-2-phenylindole, ECM extracellular matrix, FN fibronectin, PIV particle image velocimetry, GelMA gelatin methacryloyl, ColMA collagen methacryloyl, LN laminin, Kir2.1 inward rectifier potassium channel, FRJS focused rotary jet spinning

Beyond linear tissue constructs, ventricle chamber models are designed to recapitulate the macroscopic geometry and biomechanical pumping functions of the human heart. These systems enable the quantitative evaluation of pressure–volume (P–V) relationships, ejection fraction (EF), and directionally organized electrical conduction, which are essential parameters for translational and functional assessments. Early examples include engineered cardiac organoid chambers and miniature balloon catheter–based constructs that reproduce preload sensitivity and electrical responsiveness^36,37^ (Fig. 4a). Subsequent tissue-engineered ventricular models fabricated using rotational molding, three-dimensional bioprinting, or hydrogel casting have successfully replicated the anatomically relevant chamber curvature and wall architecture^38^. Recent advances in three-dimensional bioprinting have further expanded the structural and functional control of ventricular models. Notable examples include freeform reversible embedding of suspended hydrogels (FRESH)-printed collagen ventricles^39^, ventricles fabricated using fiber-reinforced gelatin-based bioinks^40^ (Fig. 4b), and the human chambered muscle pump (hChaMP) constructed using gelatin methacryloyl (GelMA) and collagen methacrylate (ColMA) bioinks^41^ (Fig. 4c). In parallel, rotary jet-spun scaffolds that reproduce helical myocardial fiber alignment have enabled ventricular constructs that exhibit apical twist and base-to-apex contraction patterns, closely resembling native cardiac mechanics^42^ (Fig. 4d). More recent studies have demonstrated that multi-lineage ventricular constructs incorporating cardiomyocytes (CMs), hiPSC-CMs, ECs, and FBs can sustain long-term functional stability and pharmacological responsiveness, supporting their application as advanced platforms for drug testing and disease modeling^43^. Collectively, these three-dimensional ventricular systems bridge the gap between conventional in vitro assays and native human myocardium, providing physiologically relevant tools for drug screening, mechanistic studies, and regenerative applications.

While engineered heart tissues and chambered ventricular models offer robust macroscopic mimicry of myocardial structure and pump function, recent advances in CO technology have enabled the bottom-up generation of miniaturized, self-organizing cardiac units that closely emulate early human cardiogenesis and cellular diversity. Therefore, the following section focuses on the emerging landscape of cardiac organoids and their potential utility in preclinical drug evaluation.

### Organoids

Organoids are self-organizing 3D cell culture systems that recapitulate the essential structural and functional features of native tissues through spontaneous spatial patterning and intrinsic cell–cell and cell–ECM interactions^44^. Cardiac organoids, primarily derived from hiPSCs, emulate the early stages of human heart development by incorporating multiple cardiac lineages, including CMs, ECs, epicardial cells, and mesenchymal stromal cells, within architecturally organized domains.

**Fig. 5:**
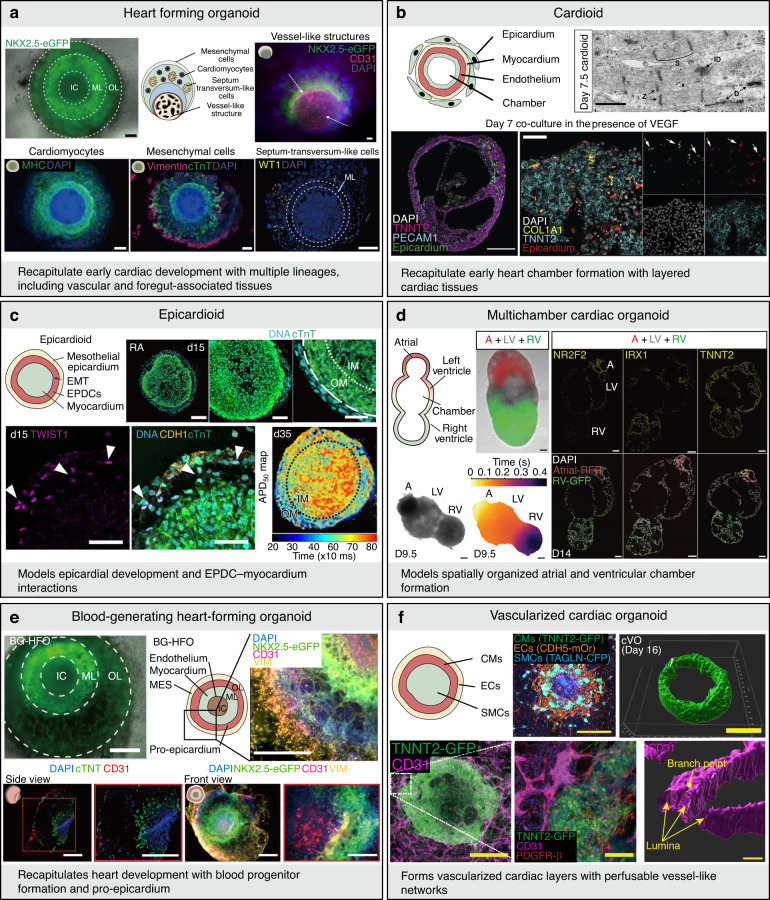
Organoid models. **a** Cardiac organoid with co-development of cardiac mesoderm and foregut endoderm, mimicking heart tube formation [scale bars = 200 µm (top-left), 100 µm (top-right), 200 µm (bottom)]. Reproduced with permission^46^. Copyright 2021, Springer Nature. **b** Self-organizing cavity-forming cardioid modeling early heart field and lumenogenesis [scale bars = 1 µm (top), 200 µm (bottom-left), 50 µm (bottom-right)]. Reproduced with permission^47^. Copyright 2021, Elsevier. **c** Epicardioid capturing epicardium formation and EMT processes [scale bars = 200 µm (top-left), 100 µm (top-middle), 50 µm (top-right), 50 µm (bottom)]. Reproduced with permission^50^. Copyright 2023, Springer Nature. **d** Multi-chamber cardiac organoid with spatially patterned atrial and ventricular domains [scale bars = 200 µm]. Reproduced with permission^51^. Copyright 2023, Elsevier. **e** Blood-generating organoid co-developing cardiac and hematopoietic lineages [scale bars = 500 µm]. Reproduced with permission^52^. Copyright 2024, Springer Nature. **f** Vascularized cardiac organoid with integrated hepatic and endothelial precursors [scale bars = 1 mm (top-left), 0.5 mm (top-right), 0.5 mm (bottom-left), 100 µm (bottom-middle), 20 µm (bottom-right)]. Reproduced with permission^55^. Copyright 2025, The American Association for the Advancement of Science. IC inner core, ML middle layer, OL outer layer, WT1 Wilms tumor 1, EPDCs epicardium-derived cells, EMT epithelial-mesenchymal transition, ID intercalated disk, RA right atrium, LV left ventricle, RV right ventricle, BG-HFO blood-generating heart-forming organoid, MES mesenchyme, CMs cardiomyocytes, ECs endothelial cells, SMCs smooth muscle cells, cVO cardiac vascularized organoid

These systems exhibit hallmark morphogenetic processes, such as lumen formation, chamber-like compartmentalization, and coordinated lineage specification, providing powerful platforms for investigating cardiogenesis, congenital heart defects, and interlineage crosstalk under physiologically relevant conditions^45^. Heart-forming organoids (HFOs) represent some of the earliest cardiac organoid models that reproduce the spatiotemporal coordination between the cardiac mesoderm and foregut endoderm, a critical interaction during primitive heart tube formation^46^ (Fig. 5a). Through biphasic WNT signaling modulation and Matrigel embedding, HFOs generate vascular and septum transversum–like tissues that mimic early cardiogenic niches.

Cardioids constitute another major milestone, as they reproduce lumenogenesis, coelomic cavity formation, and early chamber morphogenesis through intrinsic self-patterning mechanisms^47^ (Fig. 5b). The spatially segregated expression of transcription factors, such as HAND1 and NFATC1, further underscores their utility in dissecting the genetic programs governing cardiac morphogenesis. Chamber-forming heart organoids further extend this complexity by generating cavitated structures with atrial–ventricular compartmentalization and stratified myocardial and endocardial layers in the absence of externally imposed mechanical cues^48^. Elongating multi-lineage organized (EMLOC) neurocardiac gastruloids advance this paradigm by enabling the coordinated development of neuroectodermal and cardiac mesodermal tissues, facilitating investigations into early neurocardiac communication^49^. Epicardioids represent a particularly important innovation, as they recapitulate epicardial sheet formation, epithelial-to-mesenchymal transition (EMT), and dynamic interactions with the underlying myocardium and coronary progenitors, which are central to vascular development and fibrotic remodeling^50^ (Fig. 5c). In parallel, multi-chamber cardioid systems have demonstrated spatially organized atrial and ventricular domains, enabling high-resolution modeling of chamber-specific developmental abnormalities and disease phenotypes^51^ (Fig. 5d). To further enhance physiological relevance, blood-forming heart organoids have been developed that concurrently generate endocardial, myocardial, and hemogenic endothelial lineages, thereby recapitulating the integrated development of the cardiovascular and hematopoietic systems^52^ (Fig. 5e). Recently, gastruloid-based cardiac models have expanded the developmental scope of cardiac organoid technologies^53–55^. Notably, human gastruloids have been shown to self-organize into early cardiac and hepatic vascular domains, recapitulating the coordinated emergence of mesodermal and endodermal tissues during early cardiogenesis^55^ (Fig. 5f). This approach provides a powerful framework for interrogating early vascularization, inter-organ patterning, and cross-germ layer interactions in vitro, thereby bridging developmental biology and regenerative cardiac modeling.

Despite these strengths, cardiac organoids are primarily suited for modeling early cardiogenesis, congenital cardiac disorders, and multicellular developmental interactions. However, organoid morphogenesis remains inherently stochastic. Limited control over biophysical cues, therefore, constrains their utility for standardized pharmacological testing and arrhythmia risk assessment. Building on the developmental insights provided by organoid systems, MPSs have emerged as complementary platforms that offer defined microarchitectures, controlled perfusion, and programmable electrical stimulation. These features enable precise regulation of mechanical loading and real-time electrophysiological measurements, making MPSs particularly well-suited for high-throughput drug screening and cardiac safety pharmacology (Table 1).

**Table 1:** Comparison of cardiac organoid (CO) and heart-on-a-chip (HoC) systems

Aspect	Cardiac organoids (COs)	Heart-on-a-chip (HoC) systems
Formation principle	hiPSC-derived, self-organizing 3D assemblies that mimic early cardiogenesis and multicellular interactions	Engineered constructs within microfluidic chips providing defined geometry, external mechanical load, and pacing
Microenvironment control	Relies on intrinsic patterning; limited control over static biochemical and mechanical cues	External modulation of dynamic perfusion, electrical/mechanical stimulation, and pharmacokinetic gradients (via microfluidics)
Vascularization/Perfusion	Intrinsic endothelialization; generally non-perfusable networks	Integrated perfusable channels enabling active flow and multi-organ coupling
Functional readouts	Spontaneous beating, Ca²⁺ flux, morphogenesis, and contractility	Quantified electrophysiology, contractility, and metabolism under controlled stimuli
Throughput/Reproducibility	Medium throughput, variable morphogenesis	Higher reproducibility with standardized chip formats and automated assays
Representative applications	Modeling human cardiogenesis, congenital defects, fibrosis, and multicellular crosstalk	Drug screening, proarrhythmia risk assessment, metabolism-aware toxicity
Regulatory relevance	Used mainly for developmental toxicology; limited standardization	Aligned with FDA/EMA NAM initiatives and CiPA/ICH S7B-E14 cardiac safety frameworks
Long-term/Chronic modeling	Suited to developmental or regenerative timescales but with restricted quantitative endpoints	Supports repeated dosing and long-term stability for chronic toxicity analysis

*hiPSC* human induced pluripotent stem cells, *FDA* U.S. Food and Drug Administration, *EMA* European Medicines Agency, *NAM* new approach methodologies, *CiPA* comprehensive in vitro proarrhythmia assay, *ICH* International Council for Harmonisation

### Microphysiological systems (MPSs)

MPSs, often referred to as organs-on-a-chip, represent a transformative advance over traditional preclinical animal models. By reducing translational discrepancies, these systems improve clinical predictability^56^. These platforms integrate living tissues into microfluidic devices engineered to recapitulate the architecture, microenvironment, and dynamic functionality of native organs. In the cardiovascular field, cardiac-specific MPSs, particularly HoC systems, are designed to faithfully reproduce key physiological features, including electrical conduction, mechanical contraction, and pharmacological responsiveness^57^. In contrast to self-organizing COs, which are well-suited for modeling early cardiac morphogenesis and developmental disorders, HoC platforms offer precise experimental control. This includes spatiotemporal regulation of perfusion, electrical pacing, mechanical loading, and pharmacokinetic microenvironments (Table 1). HoC systems are especially advantageous for standardized drug screening, proarrhythmia risk assessment, and real-time evaluation of contractile and electrophysiological functions under well-defined conditions.

To further capture systemic physiology, multi-organ-on-a-chip (Multi-OoC) technologies have been developed to enable controlled inter-organ communication between cardiac, hepatic, and vascular tissues^58^. These integrated platforms are particularly valuable for assessing cardiotoxicity arising from both direct drug effects and indirect influences of hepatic metabolism or immune-mediated responses, thereby enhancing the physiological relevance of in vitro safety and efficacy testing^59,60^.

**Fig. 6:**
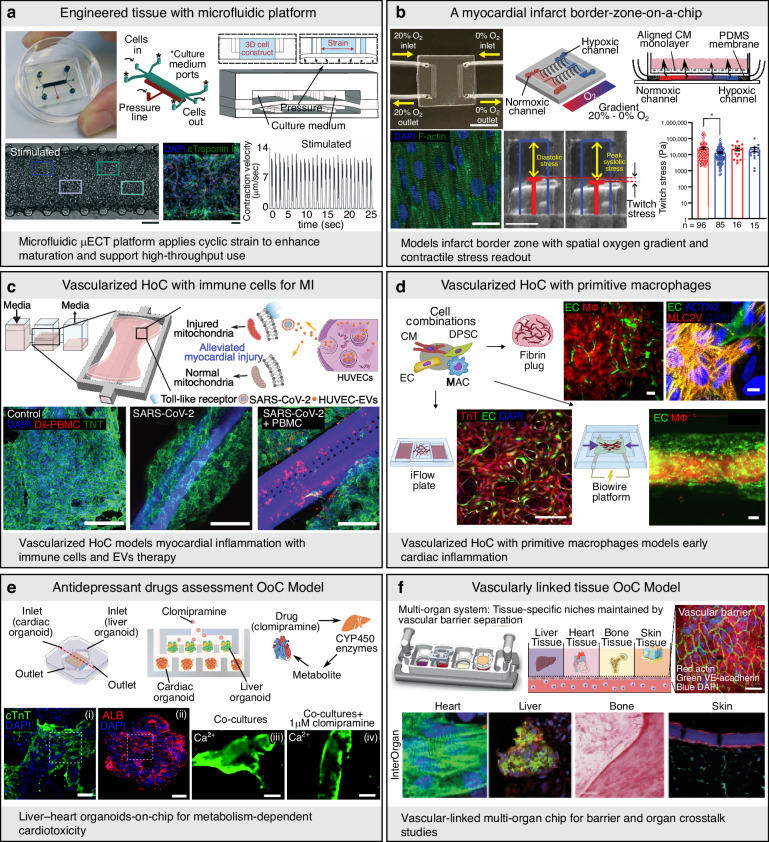
Microphysiological systems. **a** Integration of mechanical stimulation with microfluidics in micro-engineered cardiac tissues to promote tissue functionality in a beating heart-on-a-chip format [scale bars = 100 µm (left), 100 µm (right)]. Reproduced with permission^62^. Copyright 2016, Royal Society of Chemistry. **b** Creation of a myocardial infarct border-zone-on-a-chip system to simulate oxygen gradients and spatial tissue remodeling after ischemia [scale bars = 10 mm (top), 20 µm (bottom-left), 1 mm (bottom-right)]. Reproduced with permission^65^. Copyright 2022, The American Association for the Advancement of Science. **c** Immune-enhanced vascularized HoC model incorporating macrophages to recapitulate myocardial inflammation and evaluate immunomodulatory therapies [scale bar = 200 µm]. Reproduced with permission^66^. Copyright 2024, The American Association for the Advancement of Science. **d** Long-term vascularization of heart-on-a-chip achieved by incorporating primitive macrophages, promoting stable perfusion in engineered cardiac tissues [scale bars = 100 µm (top-left), 10 µm (top-right), 250 µm (bottom-left), 100 µm (bottom-right)]. Reproduced with permission^67^. Copyright 2024, Elsevier. **e** Multi-organoid chip derived from hiPSCs for assessing antidepressant-induced systemic toxicity [scale bars = 100 µm (i, ii), 200 µm (iii, iv)]. Reproduced with permission^73^. Copyright 2020, IOP Publishing. **f** A vascularly linked multi-tissue platform that connects matured organ compartments via perfusable vasculature [scale bars = 50 µm (top), 10 µm (bottom-1), 50 µm (bottom-2, 3, 4)]. Adapted with permission^74^. Copyright 2022, Springer Nature. µECT micro-engineered cardiac tissue, PDMS polydimethylsiloxane, MI myocardial infarction, EVs extracellular vesicles, DPSC dental pulp stem cell, MAC/MΦ macrophage, ALB albumin, CYP450 Cytochrome P450, OoC organ-on-a-chip, HoC heart-on-a-chip, hiPSCs human induced pluripotent stem cells, HUVECs human umbilical vein endothelial cells, PBMC peripheral blood mononuclear cell

Early MPS designs combined MTFs with microfluidic architectures to enable the real-time measurement of contractile responses under dynamic flow conditions, facilitating higher-throughput drug screening^61^. The subsequent incorporation of three-dimensional cardiac microtissues subjected to mechanical stimulation further improved cellular alignment, tissue compaction, and contractile force generation^62^ (Fig. 6a). In parallel, metabolically driven maturation strategies have enhanced hiPSC-CM function by improving calcium handling and sarcomeric organization^63^. The advent of direct laser writing (DLW) has enabled the fabrication of precision-engineered HoC devices incorporating embedded transducers for real-time electrophysiological sensing and controlled perfusion^64^. Disease modeling capabilities have also substantially advanced. For example, infarct border zone-on-a-chip systems recreate spatial oxygen gradients to simulate ischemia–reperfusion injury, revealing region-specific physiological and electrophysiological responses^65^ (Fig. 6b). The incorporation of immune components into vascularized HoC platforms has enabled mechanistic studies on inflammation-induced cardiac dysfunction. In this context, extracellular mitochondrial DNA has been identified as a trigger of pathological inflammatory signaling, which can be attenuated by exosome-based therapeutic approaches^66^ (Fig. 6c). In addition, platforms incorporating primitive macrophages have demonstrated sustained vascularization and preserved tissue function during extended culture periods^67^ (Fig. 6d). Recently, tri-culture systems comprising ECs, FBs, and hiPSC-CMs have achieved synchronized contraction and long-term functional stability under continuous perfusion, enabling robust and reproducible functional assessments^68^. Concurrently, multi-OoC platforms have progressed toward greater modularity and physiological integration in recent years. Early systems employed plug-and-play designs that allowed the customizable assembly of individual tissue modules^69^. These architectures have since evolved into integrated platforms for modeling liver–heart interactions, thereby enabling the investigation of metabolism-mediated cardiotoxicity^70,71^. More advanced designs now incorporate multiple organoids within a single perfusable circuit, permitting simultaneous drug testing across interconnected tissues and yielding comprehensive and human-relevant safety profiles^72,73^ (Fig. 6e). hiPSC-derived cardiac–hepatic chips have proven to be effective in predicting drug-induced arrhythmias and hepatotoxicity. The most advanced iterations feature vascularized and maturation-enhanced organoids interconnected by functional flow networks that simulate nutrient delivery, hormonal signaling, and systemic pathophysiology^74^ (Fig. 6f).

Advances in MPS and multi-OoC technologies are reshaping preclinical cardiac research by providing physiologically relevant, scalable human-based platforms. In parallel with these technological developments, regulatory interest has increased, with agencies such as the U.S. Food and Drug Administration (FDA) and European Medicines Agency (EMA) recognize MPS-based approaches as key New Approach Methodologies (NAMs) for cardiac safety assessment. Supported by initiatives, including the CiPA and the revised ICH S7B/E14 Q&A framework, these efforts emphasize rigorous electrophysiological validation, metabolic competence, and clinically aligned benchmark datasets. Ensuring the translational reliability of MPS platforms requires standardized multiparametric evaluation frameworks that integrate structural, functional, molecular, and metabolic analyses.

## Comprehensive assessment of cardiac models: structural, functional, molecular, and metabolic evaluations

**Fig. 7:**
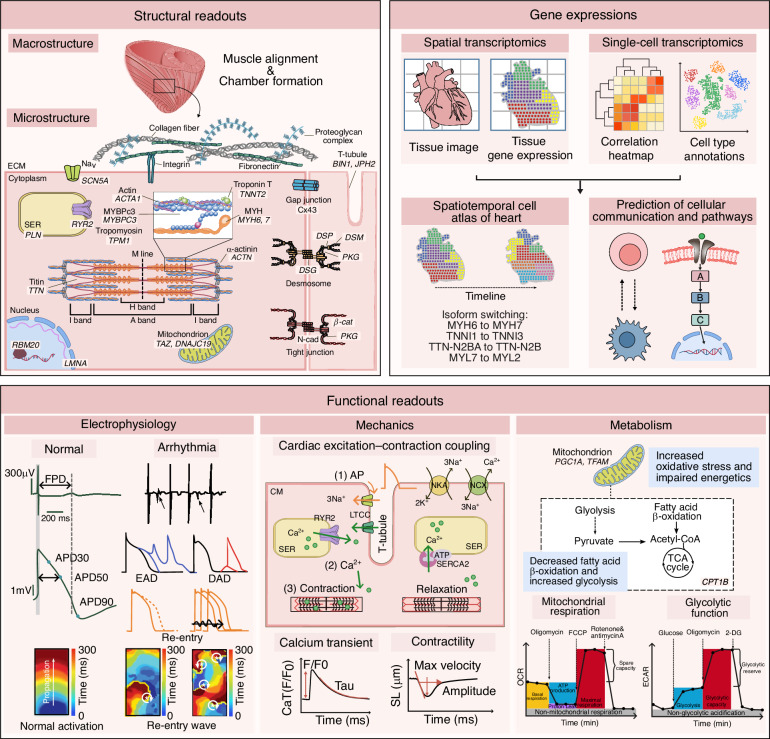
Multimodal readouts for comprehensive characterization of in vitro cardiac models. Schematic overview of structural, gene expression, and functional parameters used to evaluate engineered cardiac tissues and organoids. (Top left) Structural readouts span macrostructure (e.g., muscle alignment, chamber formation) and microstructure, including extracellular matrix composition, sarcomeric proteins, junctional complexes, and subcellular organelles. (Top right) Gene expression analysis integrates spatial and single-cell transcriptomics to map tissue architecture, spatiotemporal isoform switching, and predict intercellular communication pathways. (Bottom) Functional readouts encompass electrophysiology (e.g., action potential duration, arrhythmia patterns), mechanics (excitation–contraction coupling, calcium transients, sarcomere shortening), and metabolism (mitochondrial respiration, glycolytic activity), enabling holistic assessment of cardiac performance and disease phenotypes. SCN5A sodium voltage-gated channel alpha subunit 5 (NaV1.5), SER sarcoplasmic endoplasmic reticulum, PLN phospholamban, RYR2 ryanodine receptor 2, ACTA1 actin alpha 1, MYBPC3 myosin binding protein C, cardiac, TPM1 tropomyosin 1, MYH6 myosin heavy chain 6 (α-MHC), MYH7 myosin heavy chain 7 (β-MHC), TNNT2 troponin T type 2, TNNI3 Troponin I type 3, TTN titin, N2BA/N2B titin isoforms, LMNA lamin A/C, RBM20 RNA binding motif protein 20, TAZ tafazzin, DNAJC19 DnaJ heat shock protein family member C19, BIN1 bridging integrator 1, JPH2 Junctophilin 2, DSP desmoplakin, DSG desmoglein, DSC desmocollin, PKG plakoglobin, FPD field potential duration, APD_30_/APD_50_/APD_90_ action potential duration at 30%, 50%, 90% repolarization, EAD early afterdepolarization, DAD delayed afterdepolarization, SERCA2 sarcoplasmic reticulum Ca^2+^-ATPase 2, SL sarcomere length, CaT (F/F_0_) calcium transient, Tau decay time constant of Ca^2+^ transient, LTCC L-type calcium channel, NCX Na^+^/Ca^2+^ exchanger, NKA Na^+^/K^+^-ATPase, ATP adenosine triphosphate, PGC1A peroxisome proliferator-activated receptor gamma coactivator 1-alpha, TFAM transcription factor A, mitochondrial, CPT1B carnitine palmitoyl transferase 1B, FCCP carbonyl cyanide-p-trifluoromethoxy phenylhydrazone, 2-DG 2-deoxyglucose, ECAR extracellular acidification rate, OCR oxygen consumption rate, ECM extracellular matrix

Evaluating the translational relevance of in vitro cardiac models requires a multidimensional framework encompassing structural integrity, physiological function, molecular identity, and metabolic competence (Fig. 7, Table 2). Together, these complementary dimensions ensure that engineered cardiac tissues faithfully reflect the key properties of the native human myocardium.

**Table 2:** Assessment for each in vitro cardiac model (cont.)

In vitro models/readouts		Structural			Functional							Ref.
		Model structure	Sarcomere organization and length (SL)	Specific structures present	Electrophysiology			Mechanics			Metabolism	
					Spontaneous beating	Action Potential (AP)	Conduction velocity (CV)	Ca^2+^ handling	Contractility (Contractile force, EF, FSR, FFR etc)	Inotropic response to compounds		
Ideal model		Rod shape (150 µm in length, 20 µm in width, 15 µm in height)^162^	Organized sarcomere (Z, I, A, H, M bands), SL: 1.8–2.2 μm^163^	T-tubule, gap junction, desmosome, adherens junction, dyad, and basement membrane observed	Not observed	RMP: −90 mV, dV/dt_max_: 200 V/s, APD80: 400 ms^164^	Atrial: ~50 cm/s AV node: ~5 cm/s Bundle branches: 100–300 cm/s Purkinje system: 200–400 cm/s^165^	Peak amplitude Δ[Ca²⁺]: 0.5–1.0 μM cytosolic^166^, Time to peak: 20–30 ms^167^, Diastolic [Ca²⁺]: 100 nM^168^, SR [Ca²⁺]: 1–1.5 mM^169^	Contractile stress: ~44 kPa^170^, EF: 65.6–67.7%^171^ FSR: positive FFR: positive	Positive: isoproterenol, digoxin, dobutamine Negative: verapamil, flecainide, propranolol	Major energy source: fatty acid oxidation^172^, increased mitochondria volume fraction (~40% cell volume)^173^	^162–173^
2D	Single cell	Myocytes cultured on ECM substrate	Organized sarcomeric α-actinin^15,16,174^ SL: ~1.8 μm^15^, 1.96–2.27 μm^16^ 1.69–2.23 μm^174^	-	Observed	ET: 4.4 V/cm^175^	-	Time to peak: ~116 ms^15^	Contraction force: ~1 μN^15^, ~0.72 μN^174^ Contractile stress: ~1.60 kPa^175^, ~0.4 kPa^176^ Contraction velocity: ~1 × 10^−3^ cm/s^174^	Positive: isoproterenol^176^	-	^15,16,174–176^
	Cell pair	A pair of myocytes on micropatterned fibronectin islands	Sarcomeric organization and functional remodeling	Gap junction (Cx43), focal adhesion, ICD observed	Observed	-	-	-	Contraction force: ~0.001 μN, FSR: positive	-	-	^17^
	Monolayer	Myocytes cultured on monolayer	Organized sarcomeric α-actinin^177,178^ SL: 1.65–1.73 μm^177^	-	Observed	RMP: −80.3 mV, dV/dt_max_: 250 V/s^178^	-	Time to peak: ~250 ms^177^	Contraction force: ~13 μN^177^	-	Induced OCR (T3, maturation media)^177,178^	^177,178^
	Aligned monolayer	Muscular thin film (MTF)	Organized sarcomeric α-actinin^18,20,61,93,179^ SL: 1.94–2.07 μm^180^	Gap junction (Cx43) observed^65^	Observed	-	~ 23.7 cm/s^65^	Time to peak: 110–160 ms Decay time (t₅₀): 90–150 ms^65^	Contractile stress: 10–18 kPa^18^, 7–15 kPa^20^, 12.7 kPa^61^, ~13 kPa^65^, ~7 kPa^179^ FFR: negative^18^	Positive: isoproterenol^61^ Negative: verapamil^20,180^	Greater spare capacity (gelatin > PDMS)^18^ Reduced OCR (BTHS)^93^, increased OCR (EEV-treated)^179^	^18,20,61,65,93,179,180^
		Geometrically constrained	Organized sarcomeric α-actinin	Gap junction (Cx43), adherens junction, desmosome observed	Observed	-	15.4–21.4 cm/s	-	-	-	-	^23^
3D	EHT	Ring shape	Organized sarcomeric α-actinin (clear Z, I, A bands)^28,181^, SL: 1.49–1.83 µm^181^	Gap junction (Cx43)^28,182^, T-tubule, desmosome, adherens junction, dyad, and basement membrane observed^28^	Observed	RMP: –73 mV dV/dt_max_: 66 V/s APA: 109 mV APD90: 148 ms^28^, 230–420 ms^181^	4.1–21.4 cm/s^182^	-	Contraction force: 0.4–0.8 mN^28^ Contractile stress: 0.23 kPa^181^, 0.19–0.92 kPa^182^ FSR: positive^181,182^	Positive: isoproterenol^28,182^ Negative: nifedipine^181^	Mitochondria volume (23.9 ± 1.2%)^28^, increased OCR with reentrant waves^181^	^28,181,182^
		Pillar post cantilever	Organized sarcomeric α-actinin^29,183^ SL: 2.2 μm^29^	Gap junction (Cx43)^183^, T-tubule, desmosome observed^29^	Observed	RMP: −70.0 mV dV/dt_max_: 23.4 V/s APA: ~47 mV APD90: ~574.4 ms^29^ APD80: ~540 ms^183^	25.0 cm/s^29^, 25.8 cm/s^183^	Time to peak: 90–130 ms Decay time (t₅₀): 200 ms^183^	Contractile Stress: ~2.8 kPa^29^, ~23.2 kPa^183^, ~1.4 kPa^34^ FFR: positive^29^ FSR: positive^29^	Positive: isoproterenol^29^	Increased OCR, percent area of mitochondria: 30%^29^	^29,34,183^
		Biowire	Organized sarcomeric α-actinin^30,184,185^ SL: ~2.2 μm^184^	Gap junction (Cx43) observed^184^	Observed	RMP: −70 mV, dV/dt_max_: 25.3–108.0 mV/s APA: 95–112 mV, APD90: 110–150 ms^30^	5.6–13.0 cm/s^30^	Time to peak: 1–80 ms^30^, 96–155 ms^184^	Contraction force: ~14 µN^30^, ~40 µN^184^, ~30 µN^185^ FFR: Robust positive^30^, positive^184^	Negative: verapamil^30^	-	^30,184,185^
		Patch	Organized sarcomeric α-actinin^31,35,186^ SL: ~1.8 μm^31^	Gap junction (Cx43)^31,35^, T-tubular-like structure observed^31^	Observed	APA: ~110 mV^31^ dV/dt_max_: ~210 V/s^31^ APD80: ~300 ms^35^, ~470 ms^186^ APD90: ~280 ms^31^	~20 cm/s^35^, ~41 cm/s^31^, ~25.1 cm/s^186^	Increased Ca^2+^ amplitude (in co-culture with CMs and ECs^35^, in implanation^186^)	Contraction force: 4.6 mN^31^ Contractile stress: ~15 kPa^35^, 2.9–13.3 kPa^186^	Positive: Isoproterenol^31^	-	^31,35,186^
		Ventricle chamber	Organized sarcomeric α-actinin^37,38,40,42,43^ SL: 1.6 μm^37^, 2.03 μm^43^	Gap junction (Cx43) observed^37,41^	Observed	APD80: ~499 ms^41^ APD90: ~580 ms^37^	~8 cm/s^37^, 5.2 cm/s^38^, ~1.97 cm/s^41^, ~19.1 cm/s^42^	-	EF: 2.44%^37^, ~0.2%^38^, 5.94%^40^, 0.7–6.5%^41^,3.3%^42^ Stroke work: ~0.05 mmHg × μ^38^, 5.83 mmH_2_O∙μL^37^ Stroke volume: 4.82 mL^37^, 6.7–13.8 nL^43^ Contractile stress: 3.5 kPa^41^ FSR: positive^41^	Positive: Isoproterenol^37,38,41,43^	Upregulation of mitochondrial biogenesis gene *TFAM*^43^	^37,38,40–43^
	CO	Spheroidal self-assembled multicellular cardiac tissue	Organized sarcomeric α-actinin^46,48,51^ SL: 9.51–11.9 μm^48^	Gap junction (Cx43)^51^, ICD, desmosome observed^47^	Observed	RMP: −80 mV^46,52^, −70 mV^51^ APA: ~100 mV^46,52^, ~98 mV^51^ APD50: >200 ms^46,52^ APD80: 250–600 ms^51^ APD90: 442 ms^47^, ~320 ms^55^ dV/dt_max_: ~8 mV/ms^46^, ~5 mV/ms^52^, ~38 V/s^55^ FPD: 0.3 s^48^, ~0.25 s^55^	0.72–1.7 cm/s^51^	Time to peak: ~277 ms^55^	Contraction velocity: ~21 μm/s^47^	-	Upregulation of mitochondrial metabolism (*COX6A2, CKM, CKMT2*), fatty acid β-oxidation^48^	^46–48,51,52,55^
	MPS	Microfluidic system integrated with heart organoid or tissue (HoC)	Organized sarcomeric α-actinin^62–64^	Gap junction (Cx43)^187^, adherens junction (N-cadherin) observed^62^	Observed	ET: 0.18 V/cm^62^, 2.9–3.8 V/cm^187^, ~5.8 V/cm^67^ APD80: 260–580 ms^63^	Normalized calcium transients observed^62,187^	Time to peak: ~0.45 s^68^	Contraction force: ~38 μN^67^, 0.003 μN^64^ Contractile Stress: 4.8 kPa^63^ Contraction velocity: 7–14 μm/s^62^, 4–5.8 μm/s^187^, ~7.5 μm/s^188^	Positive: Isoproterenol^62,63,187^	Upregulation of mitochondrial protein *COX6* gene^68^	^62–64,67,68,187,188^
		Microfluidic system integrated with heart and liver organoid or tissue (MOoC)	Organized sarcomeric α-actinin^74^	-	Observed	mISI: ~1.8 s^70^ ET: 10 V/cm^74^	~15 cm/s^70^	-	Contractile stress: ~2 kPa^74^, Contraction velocity: 4–12 μm/s^69^, ~30 μm/s^73^	-	Liver organoid/tissue metabolizes prodrug/drug to cardiotoxic compound, impacting heart function^70,73^	^69,70,73,74^

*AP* action potential, *APA* action potential amplitude, *APD* action potential duration, *BTHS* Barth syndrome, *CO* cardiac organoid, *COX6* cytochrome c oxidase subunit 6, *CV* conduction velocity, *Cx43* Connexin 43, *dV/dt*_max_ maximum upstroke velocity, *EC* endothelial cell, *ECM* extracellular matrix, *EF* ejection fraction, *EHT* engineered heart tissue, *ET* excitation threshold, *EEV* endothelial Extracellular vesicle, *FFR* force-frequency relationship, *FPD* field potential duration, *FSR* Frank-Starling relationship, *HoC* heart-on-a-chip, *ICD* intercalated disk, *mISI* minimum interspike interval, *MOoC* multi-organ-on-a-chip, *MPS* microphysiological system, *MTF* muscular thin film, *OCR* oxygen consumption rate, *PKG* plakoglobin, *RMP* resting membrane potential, *SL* sarcomere length, *SR* sarcoplasmic reticulum, *TFAM* transcription factor A, mitochondrial

Structural assessment provides foundational insights into cellular architecture, focusing on sarcomere organization, intercalated disc formation, and cytoskeletal integrity in cardiomyocytes. Structural readouts span multiple length scales, ranging from macroscopic features, such as tissue alignment and chamber formation, to microscopic and subcellular characteristics, including extracellular matrix composition, sarcomeric proteins, junctional complexes, and organelle organization. Standard approaches include immunostaining for α-actinin, connexin 43 (Cx43), and desmosomal proteins such as desmoplakin (DSP) and desmoglein (DSG), combined with confocal or super-resolution microscopy to visualize myofibrillar alignment and junctional morphology^75^.

Functional evaluation centers on excitation–contraction coupling (ECC)^76^. Upon membrane depolarization, voltage-gated calcium channels are activated, triggering calcium release from the sarcoplasmic reticulum (SR) via ryanodine receptors. Cytosolic Ca²⁺ binding to troponin C initiates contraction, followed by Ca²⁺ reuptake into the SR via sarco/endoplasmic reticulum Ca²⁺-ATPase (SERCA) and extrusion through the sodium–Ca²⁺ exchanger (NCX) and plasma membrane Ca^2+^-ATPase (PMCA) to enable relaxation. These processes can be quantitatively interrogated using patch-clamp electrophysiology and multielectrode arrays, calcium imaging with indicators such as Fluo-4 or genetically encoded sensors (e.g., GCaMP), and mechanical assays, including muscular thin films, cantilever deflection, or traction force microscopy. The integration of these functional readouts provides a composite measure of cardiomyocyte maturity and electromechanical competence.

Molecular profiling adds further depth to phenotypic characterization by resolving lineage specification, maturation states, and disease-associated signatures^77^. Bulk RNA sequencing enables pathway-level analysis, whereas single-cell RNA sequencing (scRNA-seq) reveals cellular heterogeneity, rare subpopulations, and developmental trajectories^78,79^. Spatial transcriptomic approaches, such as Slide-seq and Visium, preserve tissue architecture while enabling region-specific gene expression mapping, providing insights into morphogenesis and disease progression^80^. Complementary proteomic and epigenomic analyses further elucidate regulatory networks and structural protein landscapes that are not accessible at the transcript level alone.

Metabolic assessment is equally critical because cardiomyocyte function is tightly coupled to cellular bioenergetics^81^. During maturation, cardiomyocytes undergo a metabolic transition from glycolysis to oxidative phosphorylation. This shift can be evaluated using extracellular flux analysis to quantify oxygen consumption and glycolytic activity (Seahorse OCR/ECAR), mitochondrial imaging to assess morphology, membrane potential, and reactive oxygen species (ROS) production, and metabolomic and lipidomic profiling to characterize substrate utilization and mitochondrial efficiency. Integrating multimodal datasets that encompass structural, functional, molecular, and metabolic information enables rigorous validation of in vitro cardiac platforms. These datasets include imaging, electrophysiology, transcriptomics, and bioenergetic measurements, and lay the foundation for AI-assisted models optimization and more predictive in vitro-to-in vivo translation. Advanced deep learning architectures, including multimodal transformer models and graph neural networks (GNNs), are increasingly applied to in vitro cardiac datasets. These approaches uncover latent relationships between cellular form, function, and metabolism, enabling automated phenotype classification and early detection of cardiotoxic liabilities. For example, deep learning–based imaging pipelines have enhanced high-throughput screening sensitivity by identifying subtle cardiotoxic phenotypes across libraries of more than 1200 compounds^82^. In parallel, predictive simulations and in silico modeling approaches are being used to forecast complex tissue-level dynamics and drug responses, exemplified by machine learning–optimized bioinspired cardiac constructs^83^ and computational multielectrode array models that integrate heterogeneous hiPSC-CM populations to improve the predictive accuracy of cardiac safety testing^84^. These integrative and data-driven strategies represent a critical step toward establishing robust human-relevant cardiac platforms that support next-generation precision pharmacology and cardiovascular therapies.

## Applications

### Fundamental research: human cardiogenesis and cardiomyocyte maturation

The establishment of physiologically relevant in vitro cardiac models using human induced pluripotent stem cells (hiPSCs) requires a fundamental understanding of two key biological processes: human cardiogenesis^85^ and cardiomyocyte (CM) maturation^86^. Elucidating these processes provides the developmental and functional framework necessary for generating advanced cardiac organoids, engineered heart tissues (EHTs), and disease-specific cardiac models. Moreover, such knowledge is essential for investigating the mechanisms underlying congenital and acquired heart diseases, enabling the development of targeted therapeutic strategies and advancing precision medicine in the field of cardiology. Collectively, insights into human cardiogenesis and approaches to promote hiPSC-CM maturation form the biological foundation upon which next-generation in vitro cardiac platforms are built.

#### Advances in human cardiogenesis using cardiac organoids

Cardiac organoid platforms have emerged as powerful tools for modeling the early stages of human heart development. These systems recapitulate key processes of cardiogenesis, including cardiac mesoderm induction, first and second heart field specification, chamber morphogenesis, and coordinated development of vascular lineages^87^. Their self-organizing capabilities enable controlled and reproducible investigations of complex spatial and temporal developmental processes that are otherwise difficult to study in vivo. Recent innovations have improved the resolution and mechanistic fidelity of organoid-based studies on early cardiogenesis^46,88^. These platforms have facilitated mechanistic insights into lineage specification, spatial patterning, and signaling dynamics during the development of the heart. Beyond cardiac-specific models, advanced multi-lineage organoids have been developed to emulate integrated human embryogenesis in vitro. For instance, the modulation of Wnt signaling in hiPSC aggregates has yielded organoids comprising cardiac, endodermal, and ectodermal tissues, recapitulating early foregut–heart axis interactions^49^. Gastruloid platforms support the co-development of cardiac and hepatic vasculature^55^ and simulate the simultaneous emergence of hematopoietic and cardiac lineages^52^. Additionally, epicardioid systems enable modeling of epicardial sheet formation and myocardial interactions during vascular development^50^. These advanced organoid technologies, including cardioids, gastruloids, and epicardioids, provide high-resolution in vitro platforms for studying human cardiogenesis across germ layers, offering novel insights into tissue patterning and lineage crosstalk.

#### Strategies for promoting cardiomyocyte maturation

A major limitation of hiPSC-CMs is their intrinsic structural and functional immaturity, which constrains their translational applicability^89^. Therefore, promoting cardiomyocyte maturation is essential for enhancing the physiological relevance of in vitro cardiac models. Mature hiPSC-CMs closely recapitulate adult myocardial responses, thereby enabling more accurate drug testing, improved modeling of adult-onset cardiac diseases, and enhanced integration into regenerative therapeutic strategies.

A diverse array of maturation strategies has been developed to overcome these limitations. Biomaterial-based scaffolds provide biomechanical cues that promote sarcomere alignment and contractile development^28^. In parallel, mechanical and electrical stimulation, designed to mimic physiological loading and pacing, enhances cytoskeletal organization, mitochondrial biogenesis, and calcium-handling machinery, collectively driving a more adult-like electrophysiological phenotype^29,32^. Co-culture with supportive non-myocyte populations, including fibroblasts and endothelial cells, further facilitates paracrine and juxtacrine signaling pathways that improve hiPSC-CM functional maturation and tissue-level organization^90^.

Metabolic and hormonal conditioning also play pivotal roles in the maturation of cardiomyocytes. Induction of fatty acid oxidation, coupled with hormonal modulation using triiodothyronine (T3) and glucocorticoids, has been shown to advance both electrophysiological and metabolic maturation of hiPSC-CMs^63^. These integrated maturation approaches significantly enhance key phenotypic features, including sarcomere organization, force generation, action potential duration, calcium transient kinetics, and oxidative metabolism in cardiomyocytes. Consequently, mature hiPSC-CMs exhibit substantially improved utility for disease modeling, drug screening, and translational cardiac research^75^. Ongoing investigations into cardiomyocyte maturation pathways continue to drive the development of increasingly predictive and clinically relevant in vitro cardiac platforms. Together, advances in understanding human cardiogenesis and strategies for promoting cardiomyocyte maturation establish the essential biological foundation for next-generation cardiac model systems, whose ultimate translational value lies in their ability to model human cardiovascular diseases with both physiological and pathological properties.

### Cardiac disease modeling

hiPSC-CMs have transformed cardiac disease modeling by providing patient-specific, ethically acceptable, and genetically tractable platforms for mechanistic investigations and drug screening. Unlike embryonic stem cells (ESCs), hiPSCs are reprogrammed from somatic cell sources, such as dermal fibroblasts or peripheral blood mononuclear cells (PBMCs), thereby enabling the generation of patient-derived cardiac models while avoiding ethical concerns associated with embryonic tissues^91^. This approach allows cardiac disease mechanisms to be studied in a human cellular context while preserving the unique genetic background^92^.

The utility of hiPSC-CM-based disease models is further enhanced by CRISPR/Cas9-mediated genome editing, which enables the precise insertion, correction, or deletion of pathogenic variants. When combined with isogenic control lines, this strategy allows for the direct attribution of observed phenotypes to specific genetic alterations and supports high-resolution mechanistic analyses^93^. Such genetic precision is particularly valuable for modeling monogenic cardiac disorders and evaluating mutation-targeted therapeutic strategies.

Broadly, hiPSC-based cardiac disease models can be classified into inherited and acquired models (Tables 3–7). Inherited cardiac diseases are typically modeled by generating hiPSCs from affected patients or by introducing disease-associated mutations into control lines using targeted genome editing, which recapitulates intrinsic genetic pathologies. In contrast, acquired cardiac disease models rely on the application of extrinsic stressors, such as hypoxia, inflammatory cytokines, electrical pacing, or metabolic challenge, to otherwise healthy hiPSC-CMs, mimicking environmental, metabolic, or systemic insults encountered in vivo.

**Table 3:** Inherited in vitro models of cardiac ion channelopathies

Disease		Modeling strategy: hiPSCs-CM genotype	Electrophysiological phenotype	Intervention	In vitro models	Ref
LQTS	LQTS1	Patient-derived *KCNQ1* mutation	Reduced I_Ks_ current Prolonged APD	β-blockers E-4031	2D monolayer	^94^
			Prolonged FPD	Isoproterenol	EHT	^95^
	LQTS2	Patient-derived *KCNH2* mutation	Reduced I_Kr_ Prolonged FPD	Isoproterenol	2D monolayer	^96^
			Reduced I_Kr_ Prolonged FPD EADs	Isoproterenol	EHT	^97^
		CRISPR-edited *KCNH2* mutation	Prolonged APD	E-4031 Nicorandil	HoC	^98^
	LQTS3	Patient-derived *SCN5A* mutation	Increased I_NaL_ current Prolonged APD EADs	Mexiletine	2D monolayer	^99–101^
	LQTS7 (Andersen–Tawil Syndrome)	Patient-derived *KCNJ2* mutation	Reduced I_K1_ Prolonged APD DADs	Flecainide	2D monolayer	^102^
	LQTS8 (Timothy Syndrome)	Patient-derived *CACNA1C* mutation	Prolonged I_CaL_ Prolonged APD	Roscovitine	2D monolayer	^103^
	LQTS14	Patient-derived *CALM1* mutation	Impaired CDI of I_CaL_ Prolonged APD	BAPTA-AM	2D monolayer	^104^
	LQTS15	Patient-derived *CALM2* mutation	Impaired CDI of I_CaL_ Prolonged APD	CRISPR silencing of *CALM2*	2D monolayer	^105^
	LQTS16	Transfected with the *CALM3* mutation	Impaired CDI of I_CaL_ Prolonged APD	-	2D monolayer	^106^
Triadin Knockout Syndrome (TKOS)		Patient-derived *TRDN* null mutations, CRISPR-edited *TRDN* mutation	Prolonged APD EADs, APD alternans	Protein replacement (triadin)	2D monolayer	^107^
SQTS1		Patient-derived *KCNH2* mutation	Increased I_Kr_ Shortened APD/FPD EADs	Quinidine	2D monolayer	^108^
					EHT	^97^
CPVT	CPVT1	Patient-derived *RYR2* mutation	Reentrant arrhythmias	Dantrolene	2D aligned tissue	^19^
	CPVT2	Patient-derived *CASQ2* mutation	Impaired intracellular Ca²⁺ regulation	AAV9-*CASQ2* gene therapy	2D monolayer	^109^
Brugada syndrome (BrS)		Patient-derived *SCN5A* mutation	Reduced I_Na_ current Abnormal calcium transients Beating interval variation	-	2D monolayer	^110^

*KCNQ1* Potassium Voltage-Gated Channel Subfamily Q Member 1, *I*_*Ks*_ slow delayed rectifier potassium current, *KCNH2* Potassium Voltage-Gated Channel Subfamily H Member 2, *I*_*Kr*_ rapid delayed rectifier potassium current, *APD* action potential duration, *FPD* field potential duration, *EHT* engineered heart tissue, *HoC* heart-on-a-chip, *CRISPR* clustered regularly interspaced short palindromic repeats, *EAD* early afterdepolarization, *SCN5A* sodium voltage-gated channel alpha subunit 5, *I*_*NaL*_ late sodium current, *I*_*Na*_ peak sodium current, *KCNJ2* Potassium Inwardly Rectifying Channel Subfamily J Member 2, *I*_*K1*_ inward rectifier potassium current, *DAD* delayed afterdepolarization, *CACNA1C* Calcium Voltage-Gated Channel Subunit Alpha1 C, *I*_*CaL*_ L-type calcium current, *CALM1, 2, 3* Calmodulin 1, 2, 3, *CDI* calcium-dependent inactivation, *BAPTA-AM* cell-permeable Ca²⁺ chelator, *TRDN* triadin gene, *SQTS1* Short QT Syndrome type 1, *CPVT* catecholaminergic polymorphic ventricular tachycardia, *RYR2* ryanodine receptor 2, *CASQ2* calsequestrin 2, *AAV9* adeno-associated virus serotype 9

**Table 4:** In vitro models of inherited cardiomyopathies

Disease	Modeling strategy: hiPSCs-CM genotype	Features	In vitro models	Ref.
Hypertrophic cardiomyopathy (HCM)	CRISPR-edited *MYH7* mutation	Increased contractile force Prolonged relaxation kinetics	EHT	^111^
	CRISPR-edited *MYBPC3* mutation	Increased contractile force Reduced beating frequency and relaxation time Higher spontaneous arrhythmic behavior	EHT	^112^
	Patient-derived *BRAF* mutation	Increased tissue size and twitch force Atrial natriuretic peptide gene expression	EHT	^113^
	Patient-derived *PRKAG2* mutation	Glycogen accumulation Increased twitch force Increased AMPK activity	EHT	^189^
	CRISPR-edited *ACTN2* mutation	Myofibrillar disarray Increased contractility Impaired relaxation Higher myofilament Ca^2+^ sensitivity Prolonged APD	EHT	^190^
Dilated cardiomyopathy (DCM)	Patient-derived *TNNT2* mutation	Shortened sarcomere length and sarcomere disarray Abnormal sarcomeric structure Defective calcium handling Impaired contractility	EHT	^114^
	CRISPR-edited *TNNT2* mutation	Reduced contractile force Shortened sarcomere length	EHT	^115^
	Patient-derived *TTN* mutation	Sarcomere disorganization Reduced contractile force Impaired mechano-/β-adrenergic stress responses	EHT	^116^
	CRISPR-edited *TPM1* and *VCL* mutation	Sarcomere disorganization Reduced contractile force	2D monolayer	^117^
	Patient-derived *PLN* mutation	Reduced contractile force Elevated ER stress with UPR activation	EHT	^118^
	Patient-derived *RBM20* mutation	Titin splicing abnormality Sarcomere disarray Defective calcium handling	EHT	^119^
Duchenne muscular dystrophy (DMD)	Patient-derived *DMD* mutation	ABD-1 mutation correction restored contractility and Ca²⁺ handling	2D monolayer	^120^
		Increased sensitivity to mechanical stress Defective calcium handling		^121^
		Impaired contractility (rescued by myoediting)	EHT	^122^
		Lack of initial proliferative capacity Sarcoglycan mislocalization Elevated ER stress with adipogenesis and fibrosis	CO	^123^
Arrhythmogenic cardiomyopathy (ACM)	Patient-derived *PKP2* mutation	Defective calcium handling Excessive lipogenesis Increased apoptosis	CO	^124^
		Reduced Cx43 High-rate pacing capture failure	EHT	^125^
	Patient-derived *DSP* mutation	Reduced desmoplakin Diastolic lengthening Defective calcium handling Impaired contractility	EHT	^126^
Restrictive cardiomyopathy (RCM)	Patient-derived *FLNC* mutation	Increased passive tension Impaired relaxation velocity (rescued by trequinsin)	EHT	^127^
Mitochondrial cardiomyopathy (Barth Syndrome)	Patient-derived *TAZ* mutation	Sarcomere disorganization Reduced contractile force Increased oxidative stress	2D aligned tissue	^93^

*MYH7* myosin heavy chain 7, *MYBPC3* myosin binding protein C, cardiac type, *PRKAG2* protein kinase AMP-activated non-catalytic subunit gamma 2, *AMPK* AMP-activated protein kinase, *ACTN2* alpha-actinin 2, *APD* action potential duration, *TNNT2* troponin T type 2, *TTN* titin, *TPM1* tropomyosin 1, *VCL* vinculin, *PLN* phospholamban, *ER* endoplasmic reticulum, *UPR* unfolded protein response, *RBM20* RNA binding motif protein 20, *ABD-1* actin-binding domain 1, *PKP2* plakophilin-2, *Cx43* Connexin 43, *DSP* desmoplakin, *FLNC* filamin C, *TAZ* tafazzin, *EHT* engineered heart tissue, *CO* cardiac organoid

**Table 5:** In vitro models of inherited congenital heart defect (CHD)

Disease	Modeling strategy: hiPSCs-CM genotype	Features	In vitro models	Ref.
HAND1-associated CHD	CRISPR-edited *HAND1* and *NKX2-5* KO	Deficient cavity formation	CO	^47^
ISL1-associated CHD	CRISPR-edited *ISL1* KO	Atrial and outflow tract malformation Delayed onset of contraction	CO	^51^
TBX5-associated CHD	CRISPR-edited *TBX5* KO	Impaired CM differentiation Affected heart compartments Lack of spontaneous beating	CO	^51^
FOXF1-associated CHD	CRISPR-edited *FOXF1* KO	Abnormal cavity formation and differentiation	CO	^51^
Ebstein’s anomaly	Patient-derived *NKX2-5* mutation	Ventricular-to-atrial fate shift Reduced sarcomere organization	CO	^128^

*HAND1* Heart and Neural Crest Derivatives Expressed 1, *NKX2-5* NK2 Homeobox 5, *KO* knockout, *CO* cardiac organoid, *CM* cardiomyocyte, *ISL1* ISL LIM Homeobox 1, *TBX5* T-box transcription factor 5, *FOXF1* forkhead box F1

**Table 6:** In vitro models of inherited cardiac metabolic disorders

Disease	Modeling strategy: hiPSCs-CM genotype	Features	In vitro models	Ref.
Fabry Disease	Patient-derived *GLA* mutation	Gb3 accumulation Hypertrophy Increased electrical excitability	2D monolayer	^129^
Danon Disease	Patient-derived *LAMP2* mutation	Impairment in autophagic flux Increased cell size Increased expression of natriuretic peptides Defective calcium handling Increased oxidative stress and apoptosis	2D monolayer	^130^
	CRISPR-edited *LAMP2* KO	Sarcomere disarray Increased oxidative stress CaMKIIδ overactivation Defective calcium handling		^131^
Pompe Disease	Patient-derived *GAA* mutation	Defective LAMP1/2 glycosylation and impaired N-linked synthesis	EHT	^132^

*GLA* gene encoding galactosidase alpha, *Gb3* globotriaosylceramide, *LAMP2* lysosomal-associated membrane protein type 2, *KO* knockout, *CaMKIIδ* calcium/calmodulin-dependent protein kinase II delta, *GAA* gene encoding acid alpha-glucosidase

**Table 7:** In vitro models of acquired cardiac diseases

Disease	Modeling strategy	Features	In vitro models	Ref.
Myocardial Infarction	Cryoinjury	Fibronectin and collagen accumulation Necrosis Limited cardiomyocyte proliferation	CO	^47^
	Oxygen-diffusion gradient, stimulated with noradrenaline	Pathological metabolic shift Fibrosis Defective calcium handling Impaired contractility	CO	^133^
	Chemical hypoxia induction (CoCl_2_ + glucose depletion)	Cardiac cell death Biomarker secretion Defective calcium handling Impaired contractility	CO	^134^
Myocardial Ischemia	Gas-controlled hypoxia induction	Loss of cardiomyocyte viability Disruption of cellular ultrastructure Increased angiogenic potential Increased proinflammatory cytokine secretion	CO	^135^
	Spatial oxygen gradients generated via continuous gas perfusion	Defective calcium handling Reduced contractile force Transcriptional changes Activation of inflammation	HoC	^65^
	Gas-controlled hypoxia induction	Impaired contractility Abnormal metabolic state Fibrosis Lactate normalization after reperfusion		^136^
Heart Failure/ Cardiac Remodeling	Chronic catecholamine overstimulation	Impaired contractility Hypertrophy Cardiac cell death Increased NT-proBNP secretion	EHT	^137^
Tachycardia-induced cardiomyopathy	Chronic optical tachypacing	Faster contraction kinetics Shorter action potentials Shorter effective refractory periods	EHT	^138^
Diabetic cardiomyopathy	Chemical induction of diabetic conditions	Sarcomere disorganization Defective calcium handling Impaired contractility Hypertrophy Lipid accumulation Increased oxidative stress	2D monolayer	^139^
Inflammatory cardiomyopathy (Myocarditis)	Proinflammatory stimulation (Cytokines or viral infection)	Impaired contractility Defective calcium handling Increased proinflammatory cytokine secretion	EHT	^66^

*CO* cardiac organoid, *HoC* heart-on-a-chip, *NT-proBNP* N-terminal pro B-type natriuretic peptide, *EHT* engineered heart tissue

hiPSC-derived cardiac platforms enable detailed mechanistic dissection of both inherited and acquired cardiac disorders, providing genetically defined and patient-specific models for elucidating arrhythmogenic mechanisms, disease progression, and therapeutic responses. These capabilities position hiPSC-based systems as powerful tools for precision disease modeling and the development of targeted therapies for cardiovascular diseases.

#### Modeling inherited cardiac diseases

Inherited cardiac diseases arise from pathogenic mutations affecting ion channels, sarcomeric proteins, developmental regulators, and metabolic pathways, ultimately leading to electrical instability, contractile dysfunction, and abnormal morphogenesis in the heart. Long QT syndrome (LQTS) (Table 3) is a prototypical ion channelopathy. *KCNQ1* mutations (LQTS1) reduce the slow delayed rectifier potassium current (I_Ks_), prolong action potential duration and field potential duration (APD/FPD), and induce arrhythmias that can be rescued by β-blockers or E-4031^94,95^. *KCNH2* mutations (LQTS2) similarly impair I_Kr_, prolong repolarization, and predispose patients to early afterdepolarization (EAD), partially reversed by isoproterenol^96–98^, whereas *SCN5A* mutations (LQTS3) increase I_NaL_, prolong APD, and trigger arrhythmias that are shortened by mexiletine^99–101^. Additional variants include *KCNJ2* mutations in Andersen–Tawil syndrome (LQTS7), characterized by reduced I_K1_, prolonged APD, and delayed afterdepolarization (DAD) rescued by flecainide^102^, and *CACNA1C* mutations in Timothy syndrome (LQTS8), which impair Ca²⁺ channel inactivation, leading to prolonged APD that can be improved by roscovitine^103^. Calmodulin mutations (*CALM1–3*, LQTS14–16) impair calcium-dependent inactivation (CDI) of the L-type Ca²⁺ current, prolonging APD and producing severe arrhythmogenicity, which can be reversed by Ca²^+^ buffering or CRISPR-based gene correction^104–106^. Triadin knockout syndrome (TKOS) caused by *TRDN* mutations exhibits prolonged APD, Ca²⁺ mishandling, and arrhythmic alternans that are normalized by protein replacement^107^, whereas short QT syndrome (SQTS1, *KCNH2*) demonstrates increased I_Kr_, abbreviated APD/FPD, and quinidine-sensitive EADs^97,108^. Beyond QT syndromes, catecholaminergic polymorphic ventricular tachycardia (CPVT) models carrying *RYR2* or *CASQ2* mutations show spontaneous Ca²⁺ waves and DADs under adrenergic stimulation, ameliorated by dantrolene or gene therapy^19,109^, and Brugada syndrome (*SCN5A*) models display reduced sodium currents, impaired conduction, and phase 2 reentry-like abnormalities^110^.

Inherited cardiomyopathies (Table 4) have also been extensively explored in vitro. Hypertrophic cardiomyopathy (HCM) caused by mutations in *MYH7, MYBPC3, PRKAG2, ACTN2, or BRAF* is associated with sarcomeric disarray, hypercontractility, increased Ca²⁺ sensitivity, and prolonged APD^111–113^. Dilated cardiomyopathy (DCM), modeled with mutations in *TTN, TPM1, VCL, PLN,* and *RBM20*, recapitulates sarcomere disorganization, reduced contractile force, impaired responses to mechanical and adrenergic stress, unfolded protein response activation, and defective calcium handling^114–119^, whereas correction of *ABD-1* mutations restores both contractility and Ca²⁺ regulation. Duchenne muscular dystrophy (DMD) hiPSC-CMs carrying *DMD* mutations exhibit impaired contractile function, loss of dystrophin, Ca²⁺ mishandling, and stress hypersensitivity, with partial rescue by gene-editing strategies^120–123^. Arrhythmogenic cardiomyopathy (ACM) arising from *PKP2 or DSP* mutations demonstrates reduced desmosomal integrity, lipogenesis, apoptosis, impaired conduction, and spontaneous arrhythmias, particularly under mechanical loading^124–126^. Restrictive cardiomyopathy (RCM) models with *FLNC* mutations display impaired relaxation, increased passive tension, and diastolic dysfunction^127^, whereas mitochondrial cardiomyopathy, such as Barth syndrome (*TAZ*), produces sarcomere disorganization, diminished force, oxidative stress, and contractile instability^93^.

Congenital heart defects (Table 5) have been modeled by introducing mutations into developmental regulators, such as *HAND1, TBX5, ISL1, NKX2.5*, and *FOXF1*. COs carrying these mutations reveal impaired cavity formation, atrial and outflow tract malformations, delayed contraction onset, and abnormal chamber morphogenesis, thereby highlighting the developmental origins of arrhythmogenic substrates^47,51,128^.

Inherited metabolic disorders (Table 6) also contribute to the structural and electrical dysfunction. Fabry disease (GLA) models show Gb3 accumulation, hypertrophy, Ca²⁺ mishandling, and heightened excitability^129^. Danon disease (*LAMP2*) recapitulates impaired autophagy, oxidative stress, sarcomere disarray, and hypertrophy^130,131^ and Pompe disease (GAA) reproduces glycogen overload, prolonged APD, impaired calcium kinetics, and contractile dysfunction^132^. These inherited cardiac disease models elucidate direct mechanistic links between genetic perturbations and arrhythmogenic remodeling and provide powerful in vitro platforms for testing mutation-specific therapies and advancing precision cardiology research.

#### Modeling acquired cardiac diseases

Acquired cardiac disorders (Table 7) arise from environmental stressors, systemic diseases, or physiological overload and are modeled in vitro by exposing hiPSC-derived cardiac tissues to specific extrinsic stimuli. Although these conditions are not genetically inherited, they involve maladaptive remodeling of electrophysiological and contractile properties, providing clinically relevant systems for studying arrhythmogenesis.

Myocardial infarction (MI) has been modeled using cryoinjury, which induces fibronectin and collagen deposition, cardiomyocyte necrosis, and limited regenerative responses^47^, as well as oxygen-gradient systems combined with noradrenaline exposure, which triggers metabolic reprogramming, fibrosis, and Ca²⁺ dysregulation^133^. Chemical hypoxia induced by cobalt chloride (CoCl₂) further reproduces cardiomyocyte death and arrhythmogenic electrophysiological phenotypes^134^. Myocardial ischemia has also been simulated in microfluidic platforms that generate spatial oxygen gradients under continuous perfusion, resulting in impaired calcium handling, reduced contractile force, and transcriptional activation of inflammatory programs^65,135^. Similarly, global restriction of oxygen delivery evokes contractile irregularities, metabolic acidosis, and lactate normalization upon reperfusion, closely mimicking ischemia–reperfusion dynamics^136^.

Heart failure and pathological remodeling can be induced by chronic catecholamine overstimulation in EHTs. These models exhibit progressive contractile decline, cardiomyocyte hypertrophy, and elevated natriuretic peptide secretion, consistent with maladaptive ventricular remodeling observed in vivo^137^. Tachycardia-induced cardiomyopathy has been modeled through chronic optical tachypacing of EHTs, which accelerates contraction kinetics, shortens action potential duration and refractory periods, and increases susceptibility to proarrhythmic activity^138^. Diabetic cardiomyopathy is reproduced by culturing two-dimensional monolayers under hyperglycemic and lipotoxic conditions supplemented with endothelin-1 and cortisol, leading to sarcomere disorganization, oxidative stress, lipid accumulation, cellular hypertrophy, and Ca²⁺ handling abnormalities^139^. Finally, inflammatory cardiomyopathy (myocarditis) has been modeled using proinflammatory cytokine cocktails, including IL-1β, TNF-α, and IFN-γ, or viral mimetics that induce sarcomeric loss, electrophysiological instability, and cytokine release. Notably, several pathological phenotypes can be partially rescued by endothelial cell–derived exosomes^66^. These acquired disease models capture the multifactorial nature of non-genetic cardiac pathologies and provide mechanistically relevant in vitro platforms for investigating arrhythmogenic mechanisms and evaluating therapeutic interventions.

### Cardiotoxicity evaluation and drug screening using multi-organ platforms

Cardiotoxicity remains a major concern in drug development, particularly for compounds that do not primarily target the cardiovascular system^140^. Accurate assessment of cardiotoxicity risk requires consideration of inter-organ interactions, such as the influence of hepatic metabolism on cardiac function, which are often overlooked in conventional monoculture-based in vitro systems^58^. To address these limitations, multi-organ-on-a-chip (Multi-OoC) platforms have been developed to integrate multiple tissue types, thereby enabling the physiologically relevant evaluation of systemic drug responses in human-based models.

A representative example is a liver–heart microphysiological system (MPS) incorporating hiPSC-derived cardiac and hepatic tissues, which has been used to assess the metabolism-mediated cardiotoxicity of terfenadine. In this platform, terfenadine impaired cardiac electrophysiology and contractility only in the presence of functional hepatic tissue, underscoring the critical role of metabolic coupling in accurate toxicity prediction^70^. Recently, vascularized cardiac MPS platforms have been used to evaluate the cardiotoxic effects of anticancer therapeutics on both myocardial and endothelial compartments. For instance, vandetanib, a vascular endothelial growth factor receptor (VEGFR) inhibitor, was tested using a vascularized cardiac spheroid-on-a-chip model, where it induced impaired contractile performance and rhythm disturbances^141^. Similarly, a multi-lineage heart-on-a-chip integrating cardiomyocytes, endothelial cells, and fibroblasts enabled the assessment of VEGFR/platelet-derived growth factor receptor–targeting tyrosine kinase inhibitors (VPTKIs), capturing their combined toxicity on vascular integrity and myocardial function^142^.

While these studies have substantially advanced our understanding of acute and short-term cardiotoxic effects, it is increasingly recognized that clinically relevant cardiotoxicity is predominantly chronic and cumulative in nature^143^. Most existing in vitro assays evaluate drug responses over timescales of hours to days, whereas clinical cardiotoxic manifestations often emerge only after weeks or months of continuous exposure. This temporal mismatch represents a critical gap between preclinical testing and clinical observations in cardiotoxicity assessment. Accordingly, the establishment and systematic classification of long-term cardiotoxicity models are essential for improving the predictive accuracy and translational relevance. Extended culture of hiPSC-CM–based tissues within chip-based platforms enables continuous monitoring of progressive functional deterioration, including declining contractile force, impaired calcium handling, and increasing electrophysiological instability, which are hallmarks of chronic cardiomyopathy. Maintaining tissue stability and maturation over prolonged culture periods provides further insight into metabolic adaptation, mitochondrial stress, and fibrosis-like remodeling under sustained pharmacological exposure. Consequently, future cardiotoxicity assessment strategies should incorporate long-term dynamic culture conditions and repeated dosing paradigms to more faithfully reproduce chronic exposure scenarios and bridge the gap between experimental models and clinical outcomes.

### Regenerative medicine and therapeutic potential of engineered cardiac tissues

Engineered cardiac tissues developed in vitro are increasingly being explored for regenerative medicine applications, particularly for the repair of the damaged myocardium following myocardial^144–146^. Among the most extensively investigated platforms, cardiac patches and cardiac organoids (COs) have demonstrated promising potential for transplantation-based therapies^147,148^. Cardiac patches are typically fabricated by combining hiPSC-CMs with biomaterial scaffolds such as fibrin, collagen, or synthetic polymers. Following transplantation into infarcted hearts, these constructs have shown partial restoration of contractile function and evidence of electrical coupling with the host myocardium^149^. COs, which self-organize into three-dimensional structures that recapitulate early cardiac development, have also been investigated as reparative grafts. For example, transplantation of hiPSC-derived COs into murine models of myocardial infarction has been shown to promote neovascularization and partial recovery of myocardial structure and function^150^. To further enhance post-transplantation outcomes, recent studies have emphasized prevascularization strategies, including co-culture with endothelial cells and the incorporation of proangiogenic factors, such as vascular endothelial growth factor (VEGF). These approaches accelerate anastomosis with the host vasculature, improve graft perfusion, and reduce ischemic necrosis after implantation^151,152^.

In parallel, advances in immune engineering have led to the development of hypoimmunogenic iPSC lines through the targeted deletion of human leukocyte antigen (HLA) class I and II genes and the overexpression of immunomodulatory molecules, such as programmed death-ligand 1 (PD-L1). These genetic modifications substantially reduce host immune rejection and prolong graft survival in allogeneic transplantation settings^153,154^. These developments highlight the significant translational potential of engineered cardiac tissues for myocardial repair. Nonetheless, critical challenges remain, including achieving durable graft integration, long-term electrical synchrony with the host myocardium, clinical-scale manufacturing, and regulatory feasibility, which are addressed in the following section.

## Challenges

### Technical limitations of current hiPSC-derived cardiac models

Despite substantial advances, current hiPSC-derived cardiac models remain insufficient for fully recapitulating the biological complexities of the adult human heart. A central limitation is the immaturity of hiPSC-derived cardiomyocytes (hiPSC-CMs), which retain fetal-like structural, electrophysiological, and metabolic characteristics rather than adult phenotypes^75^. Specifically, hiPSC-CMs lack mature features, including dense sarcomeric organization and transverse tubules. They also exhibit immature calcium handling and metabolic profiles that favor glycolysis over fatty acid oxidation, which are hallmarks of an early developmental state. This immaturity constrains contractile force generation, conduction velocity, and responsiveness to physiological stimuli, thereby limiting the model’s overall fidelity. Cellular heterogeneity and variability pose additional technical challenges. Even isogenic hiPSC lines derived from a single donor can display substantial line-to-line variability, resulting in inconsistent differentiation efficiency and functional readouts^155^. Moreover, experiments performed across different laboratories frequently yield divergent results because of variations in cell lines, differentiation protocols, and culture conditions. This interlaboratory and inter-donor variability undermines reproducibility and complicates cross-study comparisons^156^, posing a major obstacle to the standardization of hiPSC-CM–based platforms. Another key limitation is the incomplete recreation of the native cardiac microenvironment. Engineered cardiac constructs often rely on simplified or artificial matrices that fail to fully replicate the biochemical composition, structural hierarchy, and biomechanical properties of native extracellular matrix (ECM). Consequently, critical tissue-level features, including anisotropic fiber alignment, spatial stiffness gradients, and physiologically relevant cell–matrix signaling, are inadequately reproduced.

A particularly critical deficiency is the lack of functional vascularization in most human iPSC-derived cardiac tissues. In the absence of a perfusable capillary network, three-dimensional constructs are subject to diffusion limitations, with oxygen and nutrient transport restricted to a few hundred micrometers in the tissue. This limitation leads to hypoxic cores, metabolic stress, and cell death in thicker tissues^157^ and is widely recognized as the primary cause of necrotic core formation in cardiac organoids and engineered heart tissues. Although substantial efforts are underway to incorporate endothelial networks and angiogenic cues into cardiac organoids and heart-on-a-chip platforms, the generation of stable perfusable microvasculature remains a significant engineering challenge. Multicellular integration complicates model fidelity. Incorporating multiple cardiac cell types, including cardiomyocytes, fibroblasts, vascular cells, and immune cells, into a single construct is technically challenging, as these populations have distinct maturation timelines, metabolic requirements, and signaling dependencies. Consequently, one cell type may dominate the culture or fail to mature appropriately within the engineered niche. These microenvironmental and multicellular integration challenges often result in an electromechanical mismatch between engineered tissues and the adult myocardium.

Such mismatches have important functional consequences, particularly in translational and transplantation contexts. Tissue-engineered grafts frequently struggle to achieve robust electrical and mechanical coupling with the host myocardium, in part due to immature ion channel expression in hiPSC-CMs and mechanical incompatibility between engineered constructs and native cardiac tissue. Inadequate electrical integration can lead to conduction blocks or arrhythmogenesis, whereas mechanical mismatch arising from disparities in stiffness or force generation can impair effective force transmission. Several studies have demonstrated that immature hiPSC-CMs with limited coupling capacity can provoke arrhythmogenic events when introduced into adult cardiac tissue^158^. These technical limitations related to cellular maturity, tissue architecture, vascularization, and host integration underscore the need for continued bioengineering innovation to enhance the physiological fidelity, robustness, and translational utility of hiPSC-derived cardiac models.

### Challenges in translational research and clinical applicability

Translating hiPSC-based cardiac models from the bench to bedside introduces a distinct set of translational challenges. One of the primary hurdles is scalability, the ability to produce cardiac cells or tissues at the volume, consistency, and quality required for industrial drug screening or therapeutic applications. The large-scale production of hiPSC-CMs with uniform quality remains difficult, as current differentiation protocols are labor-intensive and subject to substantial batch-to-batch variability^159^. Consequently, the routine generation of millions of mature, functionally consistent cardiomyocytes or large, clinically relevant tissue constructs for high-throughput screening or allogeneic cell therapy remains unachieved.

The scaling of engineered cardiac tissues presents additional challenges. Although miniature cardiac organoids or microtissues are relatively straightforward to culture and manipulate, they often lack adult-level structural and functional complexity. In contrast, larger engineered tissues more closely approximate the myocardial architecture but frequently suffer from limited viability, including core necrosis and insufficient integration of supporting cell populations. These trade-offs complicate efforts to balance physiological relevance and manufacturability.

Another major translational challenge is the inter-donor and inter-line variability inherent to hiPSC-based models. Because hiPSC lines retain the genetic background of their donors, phenotypic differences, such as baseline electrophysiological properties, maturation states, and drug responsiveness, can arise even among lines derived from healthy individuals. Such variability complicates the interpretation of disease phenotypes and pharmacological responses, as it can be difficult to distinguish genuine biological effects from line-specific idiosyncrasies. Although standardization of differentiation protocols and comprehensive phenotypic characterization of each hiPSC line can mitigate some of these issues, a degree of heterogeneity remains intrinsic to the system^155^.

Regulatory acceptance is an additional bottleneck for clinical translation. Although regulatory agencies have begun to recognize hiPSC-derived cardiac models as emerging New Approach Methodologies (NAMs), particularly in the context of cardiac safety pharmacology, clear regulatory standards and validation frameworks are still evolving. Recent analyses by U.S. The Food and Drug Administration (FDA) and the Center for Drug Evaluation and Research (CDER) indicate that although submissions incorporating hiPSC-CM–based cardiotoxicity data—often derived from multielectrode array electrophysiology assays—are increasing, their influence on regulatory decision-making has thus far been limited. In many cases, submitted studies lacked a clearly defined context of use or failed to convincingly link in vitro phenotypes with clinically meaningful risks, limiting their regulatory utility. These observations underscore the need for standardized performance metrics and systematic validation of hiPSC-based cardiac assays using well-characterized human clinical data.

Finally, bridging the gap between in vitro findings and human pathophysiology remains a challenge. Reductionist cardiac models, even when engineered with high fidelity, cannot fully capture the multi-organ and systemic complexities of cardiovascular disease. Neurohumoral regulation, immune system interactions, and circulatory feedback mechanisms are largely absent in isolated cardiac tissues, making it difficult to reproduce disease manifestations driven by systemic inflammation or neuroendocrine remodeling. In addition, the immature phenotype of hiPSC-CMs limits their ability to faithfully replicate adult disease states and long-term pharmacological response. Addressing these translational barriers will require continued advances in tissue maturation, multicellular complexity, and long-term culture stability, as well as the accumulation of large-scale datasets linking hiPSC-based readouts to the patient outcomes. Until such correlations are firmly established, caution is warranted when extrapolating in vitro results to clinical settings, and hiPSC-derived cardiac platforms are likely to serve as complementary—rather than replacement—tools alongside existing preclinical models in the near term.

### Limitations in preclinical drug evaluation and safety testing

hiPSC-derived cardiac platforms play an increasingly important role in preclinical drug evaluation; however, clear limitations remain in the predictive power and scope of these assays. On the positive side, hiPSC-CMs have substantially improved the detection of certain cardiotoxic effects in vivo. For example, in the context of proarrhythmia risk assessment, multielectrode array (MEA) recordings from hiPSC-CM monolayers can reveal drug-induced alterations in repolarization and arrhythmic events, such as early afterdepolarizations, which may be overlooked in animal models. The incorporation of hiPSC-CM assays into safety pharmacology pipelines, most notably through the Comprehensive in vitro Proarrhythmia Assay (CiPA) initiative, has demonstrated more human-relevant predictions of QT prolongation and arrhythmia risk for selected compounds^160^. However, the reliability of these platforms is not uniform across all forms of cardiotoxicity. Their predictive performance is well established for compounds that directly perturb cardiac ion channels, such as hERG blockers that induce arrhythmias, but remains less validated for other mechanisms of cardiac injury. In particular, chronic structural cardiotoxicities, including anthracycline-induced cardiomyopathy, or subtle, long-term functional impairments are difficult to detect using current acute hiPSC-CM–based assays.

Most hiPSC-CM studies continue to focus on short-term endpoints, including acute changes in electrophysiology, contractility, and cell viability following drug exposure over hours to days after exposure. In contrast, clinically relevant cardiotoxicity often emerges only after weeks or months of treatment with these agents. Although modeling chronic drug responses in vitro is an active area of investigation, maintaining stable adult-like cardiomyocyte function during extended culture and repeated dosing remains technically challenging.

Several advanced three-dimensional (3D) models have begun to address this limitation by applying sustained pathological stress to induce chronic injuries. For instance, engineered heart tissues exposed to prolonged afterload or continuous neurohormonal stimulation, such as norepinephrine or transforming growth factor-β (TGF-β), exhibit progressive contractile decline, prolonged calcium transients, and arrhythmic bursts, which are characteristic of heart failure and maladaptive remodeling. While these studies demonstrate that chronic disease phenotypes can be recapitulated in vitro, such approaches are not yet routinely adopted in drug testing because of their experimental complexity and limited throughput. Consequently, reliable prediction of long-term drug effects, including fibrosis, hypertrophy, and cumulative cardiotoxicity, remains a significant challenge in hiPSC-derived systems.

Another important limitation of preclinical applications is the incomplete integration of pharmacokinetic (PK) and multi-organ factors. In vivo, cardiac drug responses are shaped by metabolism, systemic distribution, and off-target effects in the organs. To address this, multi-organ microphysiological systems (MPSs) have been developed, such as heart–liver co-culture platforms that combine hiPSC-derived cardiac tissues with liver organoids or liver-on-a-chip modules, to account for drug bioactivation and clearance. These systems can uncover cardiotoxic effects mediated by metabolites that would be missed in heart-only models. Nevertheless, constructing robust multi-organ platforms presents practical challenges, including the need to optimize tissue-specific media, match physiologically relevant flow rates and scale across organs, and enable inter-organ communication that accurately reflects human physiology. At present, no universally standardized “body-on-a-chip” platform exists, and many multi-organ systems remain bespoke, limiting their reproducibility and widespread adoption. Consequently, the routine integration of PK/pharmacodynamic considerations into in vitro cardiotoxicity testing remains uncommon.

Beyond the biological and engineering limitations, computational integration has emerged as an important complementary dimension in preclinical cardiac safety assessment. Recent advances in artificial intelligence (AI) and machine learning (ML) enable multimodal fusion of imaging, electrophysiology, calcium dynamics, and transcriptomic data to train predictive models for cardiotoxicity risk and phenotype classification^82,158,161^. In particular, self-supervised and transformer-based architectures have demonstrated superior performance over traditional ML approaches in detecting subtle morphological, electrophysiological, and conduction abnormalities in hiPSC-CM systems. These computational strategies complement organoid and microphysiological platforms by supporting automated screening, early detection of drug-induced risk signatures, and patient-specific risk stratification in drug-induced cardiotoxicity. Although widespread adoption will require standardized data pipelines and benchmarking frameworks, AI-enhanced cardiac platforms represent a promising avenue for improving predictive accuracy and reducing the reliance on animal models.

In parallel with advances in biological fidelity and computational sophistication, regulatory alignment remains essential for broader clinical and industrial applications. Recent guidance from regulatory agencies, including the U.S. FDA and the European Medicines Agency (EMA) emphasize the qualification of human-relevant NAMs and the integration of hiPSC-based assays and MPSs into cardiac safety workflows. Initiatives such as CiPA and the revised ICH S7B/E14 Q&A framework highlight the importance of standardized electrophysiological validation, reproducible functional benchmarks, and demonstrated clinical concordance for regulatory acceptance in investigational new drug (IND) submissions. Accordingly, future progress will depend not only on continued bioengineering and computational innovation but also on the development of harmonized validation pipelines, reference compound sets, and cross-laboratory reproducibility standards to support regulatory qualification and scalable deployment of these technologies.

## Conclusions and future perspectives

Human induced pluripotent stem cell (hiPSC)-derived in vitro cardiac systems represent a major advance in nonclinical drug evaluation and regenerative medicine. Given the intrinsic structure–function dependency of the heart, physiologically accurate modeling is essential for developing predictive and reliable platforms. The ability of hiPSC-based models to recapitulate human cardiac electrophysiology, contractility, and pharmacological responsiveness underscores their value as next-generation tools that address the key limitations of animal studies and immortalized cell lines. Recent advances in three-dimensional cardiac tissue engineering, including engineered heart tissues, chamber-like organoids, and heart-on-a-chip systems, have substantially enhanced the biological fidelity of these models. These constructs support long-term culture, multicellular integration, and biomechanical conditioning, enabling more accurate modeling of chronic drug responses and patient-specific phenotypes compared to conventional approaches. In safety pharmacology, hiPSC-based assays have been incorporated into the Comprehensive in vitro Proarrhythmia Assay (CiPA) framework and are increasingly recognized as Drug Development Tools by regulatory agencies.

Beyond preclinical testing, hiPSC-derived cardiac tissues are progressing toward clinical applications. Preclinical studies using engineered cardiac patches and myocardial grafts have demonstrated partial remuscularization and functional recovery in animal models of myocardial infarction, and early clinical trials of allogeneic hiPSC-based cardiac products have reported encouraging safety profiles. Together, these findings highlight the therapeutic potential of engineered cardiac tissues in patients with advanced cardiovascular diseases. Nevertheless, important challenges remain, including incomplete cardiomyocyte maturation, limited vascularization, inter-line variability, and a lack of standardized manufacturing and validation pipelines. Ongoing interdisciplinary efforts spanning biofabrication, bioreactor engineering, immune modulation, and AI-driven data integration actively address these limitations.

Looking forward, the continued integration of advanced computational and systems-level approaches with biologically engineered cardiac platforms will be essential to accelerate clinical translation. The convergence of AI-enabled multimodal analysis, large-scale biobanks, and patient-specific genomic information is expected to enhance predictive cardiotoxicity assessments and precision drug screening. In parallel, multi-organ and vascularized tissue-chip circuits will enable the modeling of systemic interactions, such as cardio-hepatic drug metabolism, immune activation, and neurohumoral regulation, thereby improving translational robustness beyond single-organ systems.

Emerging directions include: (i) multimodal deep-learning frameworks that integrate imaging, electrophysiology, calcium dynamics, and transcriptomics to detect subtle structural and functional phenotypes; (ii) generative and self-supervised AI platforms for phenotype discovery and drug-response prediction; (iii) standardized data pipelines and cloud-native cardiac data repositories to support reproducible AI benchmarking; (iv) patient-matched hiPSC models combined with gene editing and disease-specific stratification for personalized medicine; and (v) scalable Good Manufacturing Practice (GMP) workflows for clinical-grade cardiac tissues and future autologous and allogeneic cell therapies. The integration of bioengineering, stem cell biology, computational modeling, and regulatory science is establishing a next-generation ecosystem for cardiac drug development and regenerative therapies. As these technologies continue to mature, hiPSC-derived cardiac platforms are poised to complement and ultimately transform conventional nonclinical testing paradigms, ushering in an era of human-relevant, precision-engineered, and clinically actionable cardiovascular innovation.

## References

[1] Roth, G. A. et al. Global burden of cardiovascular diseases and risk factors, 1990–2019: update from the GBD 2019 study. *J. Am. Coll. Cardiol.* **76**, 2982–3021 (2020). 33309175 10.1016/j.jacc.2020.11.010 PMC7755038

[2] Waring, M. J. et al. An analysis of the attrition of drug candidates from four major pharmaceutical companies. *Nat. Rev. Drug Discov.* **14**, 475–486 (2015). 26091267 10.1038/nrd4609

[3] Hay, M., Thomas, D. W., Craighead, J. L., Economides, C. & Rosenthal, J. Clinical development success rates for investigational drugs. *Nat. Biotechnol.* **32**, 40–51 (2014). 24406927 10.1038/nbt.2786

[4] Denning, C. et al. Cardiomyocytes from human pluripotent stem cells: from laboratory curiosity to industrial biomedical platform. *Biochim. Biophys. Acta Mol. Cell Res.* **1863**, 1728–1748 (2016). 10.1016/j.bbamcr.2015.10.014 PMC5221745 26524115

[5] Olson, H. et al. Concordance of the toxicity of pharmaceuticals in humans and in animals. *Regul. Toxicol. Pharmacol.* **32**, 56–67 (2000). 11029269 10.1006/rtph.2000.1399

[6] Fermini, B. et al. A new perspective in the field of cardiac safety testing through the comprehensive in vitro proarrhythmia assay paradigm. *J. Biomol. Screen.* **21**, 1–11 (2016). 26170255 10.1177/1087057115594589

[7] Gintant, G., Sager, P. T. & Stockbridge, N. Evolution of strategies to improve preclinical cardiac safety testing. *Nat. Rev. Drug Discov.* **15**, 457–471 (2016). 26893184 10.1038/nrd.2015.34

[8] Laverty, H. et al. How can we improve our understanding of cardiovascular safety liabilities to develop safer medicines? *Br. J. Pharmacol.* **163**, 675–693 (2011). 21306581 10.1111/j.1476-5381.2011.01255.x PMC3111672

[9] Sharma, A., Wu, J. C. & Wu, S. M. Induced pluripotent stem cell-derived cardiomyocytes for cardiovascular disease modeling and drug screening. *Stem Cell Res. Ther.* **4**, 150 (2013). 24476344 10.1186/scrt380 PMC4056681

[10] Karakikes, I., Ameen, M., Termglinchan, V. & Wu, J. C. Human induced pluripotent stem cell–derived cardiomyocytes: insights into molecular, cellular, and functional phenotypes. *Circ. Res.* **117**, 80–88 (2015). 26089365 10.1161/CIRCRESAHA.117.305365 PMC4546707

[11] Lian, X. et al. Directed cardiomyocyte differentiation from human pluripotent stem cells by modulating Wnt/β-catenin signaling under fully defined conditions. *Nat. Protoc.* **8**, 162–175 (2013). 23257984 10.1038/nprot.2012.150 PMC3612968

[12] Burridge, P. W., Holmström, A. & Wu, J. C. Chemically defined culture and cardiomyocyte differentiation of human pluripotent stem cells. *Curr. Protoc. Hum. Genet.* **87**, 21.3.1-21.3.15 (2015). 10.1002/0471142905.hg2103s87 PMC4597313 26439715

[13] Sala, L., Bellin, M. & Mummery, C. L. Integrating cardiomyocytes from human pluripotent stem cells in safety pharmacology: Has the time come? *Br. J. Pharmacol.* **174**, 3749–3765 (2017). 27641943 10.1111/bph.13577 PMC5647193

[14] Mathur, A. et al. Human iPSC-based cardiac microphysiological system for drug screening applications. *Sci. Rep.* **5**, 8883 (2015). 25748532 10.1038/srep08883 PMC4352848

[15] McCain, M. L., Yuan, H., Pasqualini, F. S., Campbell, P. H. & Parker, K. K. Matrix elasticity regulates the optimal cardiac myocyte shape for contractility. *Am. J. Physiol. Heart Circ. Physiol.* **306**, H1525–H1539 (2014). 24682394 10.1152/ajpheart.00799.2013 PMC4042196

[16] Bray, M. A., Sheehy, S. P. & Parker, K. K. Sarcomere alignment is regulated by myocyte shape. *Cell Motil. Cytoskelet.* **65**, 641–651 (2008). 10.1002/cm.20290 PMC4492320 18561184

[17] McCain, M. L., Lee, H., Aratyn-Schaus, Y., Kléber, A. G. & Parker, K. K. Cooperative coupling of cell-matrix and cell–cell adhesions in cardiac muscle. *Proc. Natl. Acad. Sci. USA* **109**, 9881–9886 (2012). 22675119 10.1073/pnas.1203007109 PMC3382528

[18] McCain, M. L., Agarwal, A., Nesmith, H. W., Nesmith, A. P. & Parker, K. K. Micromolded gelatin hydrogels for extended culture of engineered cardiac tissues. *Biomaterials* **35**, 5462–5471 (2014). 24731714 10.1016/j.biomaterials.2014.03.052 PMC4057039

[19] Park, S.-J. et al. Insights into the pathogenesis of catecholaminergic polymorphic ventricular tachycardia from engineered human heart tissue. *Circulation* **140**, 390–404 (2019). 31311300 10.1161/CIRCULATIONAHA.119.039711 PMC6750809

[20] Lind, J. U. et al. Instrumented cardiac microphysiological devices via multimaterial three-dimensional printing. *Nat. Mater.* **16**, 303–308 (2017). 27775708 10.1038/nmat4782 PMC5321777

[21] Batalov, I., Jallerat, Q., Kim, S., Bliley, J. & Feinberg, A. W. Engineering aligned human cardiac muscle using developmentally inspired fibronectin micropatterns. *Sci. Rep.* **11**, 11502 (2021). 34075068 10.1038/s41598-021-87550-y PMC8169656

[22] Lee, K. Y. et al. An autonomously swimming biohybrid fish designed with human cardiac biophysics. *Science* **375**, 639–647 (2022). 35143298 10.1126/science.abh0474 PMC8939435

[23] Kim, S. L. et al. Spatiotemporal cell junction assembly in human iPSC-CM models of arrhythmogenic cardiomyopathy. *Stem Cell Rep.* **18**, 1811–1826 (2023). 10.1016/j.stemcr.2023.07.005 PMC10545490 37595583

[24] Colatsky, T. et al. The comprehensive in vitro proarrhythmia assay (CiPA) initiative—update on progress. *J. Pharmacol. Toxicol. Methods* **81**, 15–20 (2016). 27282641 10.1016/j.vascn.2016.06.002

[25] Burridge, P. W. et al. Human induced pluripotent stem cell-derived cardiomyocytes recapitulate the predilection of breast cancer patients to doxorubicin-induced cardiotoxicity. *Nat. Med.* **22**, 547–556 (2016). 27089514 10.1038/nm.4087 PMC5086256

[26] Huang, G. et al. Titin-truncating variants in hiPSC cardiomyocytes induce pathogenic proteinopathy and sarcomere defects with preserved core contractile machinery. *Stem Cell Rep.* **18**, 220–236 (2023). 10.1016/j.stemcr.2022.11.008 PMC9860080 36525964

[27] Ketabat, F., Alcorn, J., Kelly, M. E., Badea, I. & Chen, X. Cardiac tissue engineering: a journey from scaffold fabrication to in vitro characterization. *Small Sci.* **4**, 2400079 (2024). 40212070 10.1002/smsc.202400079 PMC11935279

[28] Zimmermann, W.-H. et al. Tissue engineering of a differentiated cardiac muscle construct. *Circ. Res.* **90**, 223–230 (2002). 11834716 10.1161/hh0202.103644

[29] Ronaldson-Bouchard, K. et al. Advanced maturation of human cardiac tissue grown from pluripotent stem cells. *Nature* **556**, 239–243 (2018). 29618819 10.1038/s41586-018-0016-3 PMC5895513

[30] Zhao, Y. et al. A platform for generation of chamber-specific cardiac tissues and disease modeling. *Cell* **176**, 913–927.e918 (2019). 30686581 10.1016/j.cell.2018.11.042 PMC6456036

[31] Querdel, E. et al. Human engineered heart tissue patches remuscularize the injured heart in a dose-dependent manner. *Circulation* **143**, 1991–2006 (2021). 33648345 10.1161/CIRCULATIONAHA.120.047904 PMC8126500

[32] Pretorius, D., Kahn-Krell, A. M., LaBarge, W. C., Lou, X. & Zhang, J. Engineering of thick human functional myocardium via static stretching and electrical stimulation. *Iscience* **25**, 103824 (2022). 10.1016/j.isci.2022.103824 PMC8873611 35243219

[33] Jebran, A.-F. et al. Engineered heart muscle allografts for heart repair in primates and humans. *Nature* **639**, 503–511 (2025). 39880949 10.1038/s41586-024-08463-0 PMC11903342

[34] Lock, R. I. et al. Macrophages enhance contractile force in iPSC-derived human engineered cardiac tissue. *Cell Rep.* **43**, 114302 (2024). 10.1016/j.celrep.2024.114302 PMC11254687 38824644

[35] Li, H. et al. In vitro vascularization improves in vivo functionality of human engineered cardiac tissues. *Acta Biomater.* **211**, 61–73 (2026). 10.1016/j.actbio.2024.11.014 PMC12064791 39528062

[36] Lee, E. J., Kim, D. E., Azeloglu, E. U. & Costa, K. D. Engineered cardiac organoid chambers: toward a functional biological model ventricle. *Tissue Eng. Part A* **14**, 215–225 (2008). 18333774 10.1089/tea.2007.0351

[37] Li, R. A. et al. Bioengineering an electro-mechanically functional miniature ventricular heart chamber from human pluripotent stem cells. *Biomaterials* **163**, 116–127 (2018). 29459321 10.1016/j.biomaterials.2018.02.024 PMC6561506

[38] MacQueen, L. A. et al. A tissue-engineered scale model of the heart ventricle. *Nat. Biomed. Eng.* **2**, 930–941 (2018). 31015723 10.1038/s41551-018-0271-5 PMC6774355

[39] Lee, A. et al. 3D bioprinting of collagen to rebuild components of the human heart. *Science* **365**, 482–487 (2019). 31371612 10.1126/science.aav9051

[40] Choi, S. et al. Fibre-infused gel scaffolds guide cardiomyocyte alignment in 3D-printed ventricles. *Nat. Mater.* **22**, 1039–1046 (2023). 37500957 10.1038/s41563-023-01611-3 PMC10686196

[41] Kupfer, M. E. et al. In situ expansion, differentiation, and electromechanical coupling of human cardiac muscle in a 3D bioprinted, chambered organoid. *Circ. Res.* **127**, 207–224 (2020). 32228120 10.1161/CIRCRESAHA.119.316155 PMC8210857

[42] Chang, H. et al. Recreating the heart’s helical structure-function relationship with focused rotary jet spinning. *Science* **377**, 180–185 (2022). 35857545 10.1126/science.abl6395 PMC10077766

[43] Kuckelkorn, C. et al. Engineered in vitro multi-cell type ventricle model generates long-term pulsatile flow and modulates cardiac output in response to cardioactive drugs. *Adv. Healthc. Mater.* **14**, 2403897 (2025). 39943918 10.1002/adhm.202403897 PMC12004430

[44] Lancaster, M. A. & Knoblich, J. A. Organogenesis in a dish: modeling development and disease using organoid technologies. *Science* **345**, 1247125 (2014). 25035496 10.1126/science.1247125

[45] Kim, H., Kamm, R. D., Vunjak-Novakovic, G. & Wu, J. C. Progress in multicellular human cardiac organoids for clinical applications. *Cell Stem Cell* **29**, 503–514 (2022). 35395186 10.1016/j.stem.2022.03.012 PMC9352318

[46] Drakhlis, L. et al. Human heart-forming organoids recapitulate early heart and foregut development. *Nat. Biotechnol.* **39**, 737–746 (2021). 33558697 10.1038/s41587-021-00815-9 PMC8192303

[47] Hofbauer, P. et al. Cardioids reveal self-organizing principles of human cardiogenesis. *Cell* **184**, 3299–3317.e3222 (2021). 34019794 10.1016/j.cell.2021.04.034

[48] Lee, S.-G. et al. Generation of human iPSCs derived heart organoids structurally and functionally similar to heart. *Biomaterials* **290**, 121860 (2022). 36274511 10.1016/j.biomaterials.2022.121860

[49] Olmsted, Z. T. & Paluh, J. L. A combined human gastruloid model of cardiogenesis and neurogenesis. *Iscience* **25**, 104486 (2022). 10.1016/j.isci.2022.104486 PMC9198845 35721464

[50] Meier, A. B. et al. Epicardioid single-cell genomics uncovers principles of human epicardium biology in heart development and disease. *Nat. Biotechnol.* **41**, 1787–1800 (2023). 37012447 10.1038/s41587-023-01718-7 PMC10713454

[51] Schmidt, C. et al. Multi-chamber cardioids unravel human heart development and cardiac defects. *Cell* **186**, 5587–5605.e5527 (2023). 38029745 10.1016/j.cell.2023.10.030

[52] Dardano, M. et al. Blood-generating heart-forming organoids recapitulate co-development of the human haematopoietic system and the embryonic heart. *Nat. Cell Biol.* **26**, 1984–1996 (2024). 39379702 10.1038/s41556-024-01526-4 PMC11567889

[53] Rossi, G. et al. Capturing cardiogenesis in gastruloids. *Cell Stem Cell* **28**, 230–240.e236 (2021). 33176168 10.1016/j.stem.2020.10.013 PMC7867643

[54] Silva, A. C. et al. Co-emergence of cardiac and gut tissues promotes cardiomyocyte maturation within human iPSC-derived organoids. *Cell Stem Cell* **28**, 2137–2152.e2136 (2021). 34861147 10.1016/j.stem.2021.11.007

[55] Abilez, O. J. et al. Gastruloids enable modeling of the earliest stages of human cardiac and hepatic vascularization. *Science* **388**, eadu9375 (2025). 40472086 10.1126/science.adu9375 PMC12815606

[56] Wang, K. et al. Microphysiological systems: design, fabrication, and applications. *ACS Biomater. Sci. Eng.* **6**, 3231–3257 (2020). 33204830 10.1021/acsbiomaterials.9b01667 PMC7668566

[57] Butler, D. & Reyes, D. R. Heart-on-a-chip systems: disease modeling and drug screening applications. *Lab Chip* **24**, 1494–1528 (2024). 38318723 10.1039/d3lc00829k

[58] Skardal, A., Shupe, T. & Atala, A. Organoid-on-a-chip and body-on-a-chip systems for drug screening and disease modeling. *Drug Discov. Today* **21**, 1399–1411 (2016). 27422270 10.1016/j.drudis.2016.07.003 PMC9039871

[59] Ewart, L. et al. Navigating tissue chips from development to dissemination: a pharmaceutical industry perspective. *Exp. Biol. Med.* **242**, 1579–1585 (2017). 10.1177/1535370217715441 PMC5661770 28622731

[60] Sung, J. H. et al. Recent advances in body-on-a-chip systems. *Anal. Chem.* **91**, 330–351 (2018). 30472828 10.1021/acs.analchem.8b05293 PMC6687466

[61] Agarwal, A., Goss, J. A., Cho, A., McCain, M. L. & Parker, K. K. Microfluidic heart on a chip for higher throughput pharmacological studies. *Lab Chip* **13**, 3599–3608 (2013). 23807141 10.1039/c3lc50350j PMC3786400

[62] Marsano, A. et al. Beating heart on a chip: a novel microfluidic platform to generate functional 3D cardiac microtissues. *Lab Chip* **16**, 599–610 (2016). 26758922 10.1039/c5lc01356a

[63] Huebsch, N. et al. Metabolically driven maturation of human-induced-pluripotent-stem-cell-derived cardiac microtissues on microfluidic chips. *Nat. Biomed. Eng.* **6**, 372–388 (2022). 35478228 10.1038/s41551-022-00884-4 PMC10344596

[64] Jayne, R. K. et al. Direct laser writing for cardiac tissue engineering: a microfluidic heart on a chip with integrated transducers. *Lab Chip* **21**, 1724–1737 (2021). 33949395 10.1039/d0lc01078b

[65] Rexius-Hall, M. L. et al. A myocardial infarct border-zone-on-a-chip demonstrates distinct regulation of cardiac tissue function by an oxygen gradient. *Sci. Adv.* **8**, eabn7097 (2022). 36475790 10.1126/sciadv.abn7097 PMC9728975

[66] Lu, R. X. Z. et al. Cardiac tissue model of immune-induced dysfunction reveals the role of free mitochondrial DNA and the therapeutic effects of exosomes. *Sci. Adv.* **10**, eadk0164 (2024). 38536913 10.1126/sciadv.adk0164 PMC10971762

[67] Landau, S. et al. Primitive macrophages enable long-term vascularization of human heart-on-a-chip platforms. *Cell Stem Cell* **31**, 1222–1238.e1210 (2024). 38908380 10.1016/j.stem.2024.05.011 PMC11297673

[68] Liu, Y. et al. Human heart-on-a-chip microphysiological system comprising endothelial cells, fibroblasts, and iPSC-derived cardiomyocytes. *Sci. Rep.* **14**, 18063 (2024). 39117679 10.1038/s41598-024-68275-0 PMC11310341

[69] Loskill, P., Marcus, S. G., Mathur, A., Reese, W. M. & Healy, K. E. μOrgano: a Lego®-like plug & play system for modular multi-organ-chips. *PLoS ONE* **10**, e0139587 (2015). 26440672 10.1371/journal.pone.0139587 PMC4595286

[70] Oleaga, C. et al. Investigation of the effect of hepatic metabolism on off-target cardiotoxicity in a multi-organ human-on-a-chip system. *Biomaterials* **182**, 176–190 (2018). 30130706 10.1016/j.biomaterials.2018.07.062 PMC6126670

[71] Rajan, S. A. P. et al. Probing prodrug metabolism and reciprocal toxicity with an integrated and humanized multi-tissue organ-on-a-chip platform. *Acta Biomater.* **106**, 124–135 (2020). 32068138 10.1016/j.actbio.2020.02.015 PMC11083435

[72] Skardal, A. et al. Drug compound screening in single and integrated multi-organoid body-on-a-chip systems. *Biofabrication* **12**, 025017 (2020). 32101533 10.1088/1758-5090/ab6d36

[73] Yin, F. et al. HiPSC-derived multi-organoids-on-chip system for safety assessment of antidepressant drugs. *Lab Chip* **21**, 571–581 (2021). 33319899 10.1039/d0lc00921k

[74] Ronaldson-Bouchard, K. et al. A multi-organ chip with matured tissue niches linked by vascular flow. *Nat. Biomed. Eng.* **6**, 351–371 (2022). 35478225 10.1038/s41551-022-00882-6 PMC9250010

[75] Karbassi, E. et al. Cardiomyocyte maturation: advances in knowledge and implications for regenerative medicine. *Nat. Rev. Cardiol.* **17**, 341–359 (2020). 32015528 10.1038/s41569-019-0331-x PMC7239749

[76] Bers, D. M. Cardiac excitation–contraction coupling. *Nature* **415**, 198–205 (2002). 11805843 10.1038/415198a

[77] Litviňuková, M. et al. Cells of the adult human heart. *Nature* **588**, 466–472 (2020). 32971526 10.1038/s41586-020-2797-4 PMC7681775

[78] Tucker, N. R. et al. Transcriptional and cellular diversity of the human heart. *Circulation* **142**, 466–482 (2020). 32403949 10.1161/CIRCULATIONAHA.119.045401 PMC7666104

[79] Miranda, A. M. et al. Single-cell transcriptomics for the assessment of cardiac disease. *Nat. Rev. Cardiol.* **20**, 289–308 (2023). 36539452 10.1038/s41569-022-00805-7

[80] Farah, E. N. et al. Spatially organized cellular communities form the developing human heart. *Nature* **627**, 854–864 (2024). 38480880 10.1038/s41586-024-07171-z PMC10972757

[81] Bornstein, M. R., Tian, R. & Arany, Z. Human cardiac metabolism. *Cell Metab.* **36**, 1456–1481 (2024). 38959861 10.1016/j.cmet.2024.06.003 PMC11290709

[82] Grafton, F. et al. Deep learning detects cardiotoxicity in a high-content screen with induced pluripotent stem cell-derived cardiomyocytes. *eLife* **10**, e68714 (2021). 34338636 10.7554/eLife.68714 PMC8367386

[83] Zimmerman, J. F. et al. Bioinspired design of a tissue-engineered ray with machine learning. *Sci. Robot.* **10**, eadr6472 (2025). 39937888 10.1126/scirobotics.adr6472

[84] Botti, S., Krause, R. & Pavarino, L. F. In silico modelling of multi-electrode arrays for enhancing cardiac drug testing on hiPSC-CM heterogeneous tissues. *J. Physiol.*10.1113/JP287276 (2025). 10.1113/JP287276 PMC13628577 40349301

[85] Li, Y. et al. The molecular mechanisms of cardiac development and related diseases. *Signal Transduct. Target. Ther.* **9**, 368 (2024). 39715759 10.1038/s41392-024-02069-8 PMC11666744

[86] Singh, B. N. et al. Proliferation and maturation: janus and the art of cardiac tissue engineering. *Circ. Res.* **132**, 519–540 (2023). 36795845 10.1161/CIRCRESAHA.122.321770 PMC9943541

[87] Ni, B., Ye, L., Zhang, Y., Hu, S. & Lei, W. Advances in humanoid organoid-based research on inter-organ communications during cardiac organogenesis and cardiovascular diseases. *J. Transl. Med.* **23**, 380 (2025). 40156006 10.1186/s12967-025-06381-x PMC11951738

[88] Lewis-Israeli, Y. R. et al. Self-assembling human heart organoids for the modeling of cardiac development and congenital heart disease. *Nat. Commun.* **12**, 5142 (2021). 34446706 10.1038/s41467-021-25329-5 PMC8390749

[89] Li, W. et al. Comprehensive promotion of iPSC-CM maturation by integrating metabolic medium with nanopatterning and electrostimulation. *Nat. Commun.* **16**, 2785 (2025). 40118846 10.1038/s41467-025-58044-6 PMC11928738

[90] Giacomelli, E. et al. Human-iPSC-derived cardiac stromal cells enhance maturation in 3D cardiac microtissues and reveal non-cardiomyocyte contributions to heart disease. *Cell Stem Cell* **26**, 862–879.e811 (2020). 32459996 10.1016/j.stem.2020.05.004 PMC7284308

[91] Takahashi, K. et al. Induction of pluripotent stem cells from adult human fibroblasts by defined factors. *Cell* **131**, 861–872 (2007). 18035408 10.1016/j.cell.2007.11.019

[92] Musunuru, K. et al. Induced pluripotent stem cells for cardiovascular disease modeling and precision medicine: a scientific statement from the American Heart Association. *Circ. Genom. Precis. Med.* **11**, e000043 (2018). 29874173 10.1161/HCG.0000000000000043 PMC6708586

[93] Wang, G. et al. Modeling the mitochondrial cardiomyopathy of Barth syndrome with induced pluripotent stem cell and heart-on-chip technologies. *Nat. Med.* **20**, 616–623 (2014). 24813252 10.1038/nm.3545 PMC4172922

[94] Moretti, A. et al. Patient-specific induced pluripotent stem-cell models for long-QT syndrome. *N. Engl. J. Med.* **363**, 1397–1409 (2010). 20660394 10.1056/NEJMoa0908679

[95] Giacomelli, E., Sala, L., Ward-van Oostwaard, D. & Bellin, M. Cardiac microtissues from human pluripotent stem cells recapitulate the phenotype of long-QT syndrome. *Biochem. Biophys. Res. Commun.* **572**, 118–124 (2021). 34364290 10.1016/j.bbrc.2021.07.068

[96] Chang, Y. et al. hERG-deficient human embryonic stem cell-derived cardiomyocytes for modelling QT prolongation. *Stem Cell Res. Ther.* **12**, 278 (2021). 33962658 10.1186/s13287-021-02346-1 PMC8103639

[97] Goldfracht, I. et al. Engineered heart tissue models from hiPSC-derived cardiomyocytes and cardiac ECM for disease modeling and drug testing applications. *Acta Biomater.* **92**, 145–159 (2019). 31075518 10.1016/j.actbio.2019.05.016

[98] Veldhuizen, J. et al. Modeling long QT syndrome type 2 on-a-chip via in-depth assessment of isogenic gene-edited 3D cardiac tissues. *Sci. Adv.* **8**, eabq6720 (2022). 36525500 10.1126/sciadv.abq6720 PMC9757749

[99] McKeithan, W. L. et al. Reengineering an antiarrhythmic drug using patient hiPSC cardiomyocytes to improve therapeutic potential and reduce toxicity. *Cell Stem Cell* **27**, 813–821.e816 (2020). 32931730 10.1016/j.stem.2020.08.003 PMC7655512

[100] Malan, D. et al. Human iPS cell model of type 3 long QT syndrome recapitulates drug-based phenotype correction. *Basic Res. Cardiol.* **111**, 14 (2016). 26803770 10.1007/s00395-016-0530-0 PMC4724360

[101] Ma, D. et al. Modeling type 3 long QT syndrome with cardiomyocytes derived from patient-specific induced pluripotent stem cells. *Int. J. Cardiol.* **168**, 5277–5286 (2013). 23998552 10.1016/j.ijcard.2013.08.015

[102] Kuroda, Y. et al. Flecainide ameliorates arrhythmogenicity through NCX flux in Andersen-Tawil syndrome-iPS cell-derived cardiomyocytes. *Biochem. Biophys. Rep.* **9**, 245–256 (2017). 28956012 10.1016/j.bbrep.2017.01.002 PMC5614591

[103] Yazawa, M. et al. Using induced pluripotent stem cells to investigate cardiac phenotypes in Timothy syndrome. *Nature* **471**, 230–234 (2011). 21307850 10.1038/nature09855 PMC3077925

[104] Rocchetti, M. et al. Elucidating arrhythmogenic mechanisms of long-QT syndrome CALM1-F142L mutation in patient-specific induced pluripotent stem cell-derived cardiomyocytes. *Cardiovasc. Res.* **113**, 531–541 (2017). 28158429 10.1093/cvr/cvx006

[105] Limpitikul, W. B. et al. A precision medicine approach to the rescue of function on malignant calmodulinopathic long-QT syndrome. *Circ. Res.* **120**, 39–48 (2017). 27765793 10.1161/CIRCRESAHA.116.309283 PMC5516949

[106] Wren, L. M. et al. Genetic mosaicism in calmodulinopathy. *Circ. Genom. Precis. Med.* **12**, e002581 (2019). 10.1161/CIRCGEN.119.002581 PMC6751013 31454269

[107] Clemens, D. J. et al. Cellular and electrophysiological characterization of triadin knockout syndrome using induced pluripotent stem cell-derived cardiomyocytes. *Stem Cell Rep.* **18**, 1075–1089 (2023). 10.1016/j.stemcr.2023.04.005 PMC10202692 37163978

[108] El-Battrawy, I. et al. Modeling short QT syndrome using human-induced pluripotent stem cell–derived cardiomyocytes. *J. Am. Heart Assoc.* **7**, e007394 (2018). 29574456 10.1161/JAHA.117.007394 PMC5907581

[109] Lodola, F. et al. Adeno-associated virus-mediated CASQ2 delivery rescues phenotypic alterations in a patient-specific model of recessive catecholaminergic polymorphic ventricular tachycardia. *Cell Death Dis.* **7**, e2393–e2393 (2016). 27711080 10.1038/cddis.2016.304 PMC5133973

[110] Liang, P. et al. Patient-specific and genome-edited induced pluripotent stem cell–derived cardiomyocytes elucidate single-cell phenotype of Brugada syndrome. *J. Am. Coll. Cardiol.* **68**, 2086–2096 (2016). 27810048 10.1016/j.jacc.2016.07.779 PMC5373649

[111] Cohn, R. et al. A contraction stress model of hypertrophic cardiomyopathy due to sarcomere mutations. *Stem Cell Rep.* **12**, 71–83 (2019). 10.1016/j.stemcr.2018.11.015 PMC6335568 30554920

[112] Flenner, F. et al. Translational investigation of electrophysiology in hypertrophic cardiomyopathy. *J. Mol. Cell. Cardiol.* **157**, 77–89 (2021). 33957110 10.1016/j.yjmcc.2021.04.009 PMC8320769

[113] Cashman, T. J., Josowitz, R., Johnson, B. V., Gelb, B. D. & Costa, K. D. Human engineered cardiac tissues created using induced pluripotent stem cells reveal functional characteristics of BRAF-mediated hypertrophic cardiomyopathy. *PLoS ONE* **11**, e0146697 (2016). 26784941 10.1371/journal.pone.0146697 PMC4718533

[114] Dai, Y. et al. Troponin destabilization impairs sarcomere-cytoskeleton interactions in iPSC-derived cardiomyocytes from dilated cardiomyopathy patients. *Sci. Rep.* **10**, 209 (2020). 31937807 10.1038/s41598-019-56597-3 PMC6959358

[115] Pettinato, A. M. et al. Development of a cardiac sarcomere functional genomics platform to enable scalable interrogation of human TNNT2 variants. *Circulation* **142**, 2262–2275 (2020). 33025817 10.1161/CIRCULATIONAHA.120.047999 PMC7722228

[116] Hinson, J. T. et al. Titin mutations in iPS cells define sarcomere insufficiency as a cause of dilated cardiomyopathy. *Science* **349**, 982–986 (2015). 26315439 10.1126/science.aaa5458 PMC4618316

[117] Deacon, D. C. et al. Combinatorial interactions of genetic variants in human cardiomyopathy. *Nat. Biomed. Eng.* **3**, 147–157 (2019). 30923642 10.1038/s41551-019-0348-9 PMC6433174

[118] Feyen, D. A. et al. Unfolded protein response as a compensatory mechanism and potential therapeutic target in PLN R14del cardiomyopathy. *Circulation* **144**, 382–392 (2021). 33928785 10.1161/CIRCULATIONAHA.120.049844 PMC8667423

[119] Streckfuss-Bömeke, K. et al. Severe DCM phenotype of patient harboring RBM20 mutation S635A can be modeled by patient-specific induced pluripotent stem cell-derived cardiomyocytes. *J. Mol. Cell. Cardiol.* **113**, 9–21 (2017). 28941705 10.1016/j.yjmcc.2017.09.008

[120] Kyrychenko, V. et al. Functional correction of dystrophin actin binding domain mutations by genome editing. *JCI insight* **2**, e95918 (2017). 28931764 10.1172/jci.insight.95918 PMC5621913

[121] Guan, X. et al. Dystrophin-deficient cardiomyocytes derived from human urine: new biologic reagents for drug discovery. *Stem Cell Res.* **12**, 467–480 (2014). 24434629 10.1016/j.scr.2013.12.004 PMC3966181

[122] Long, C. et al. Correction of diverse muscular dystrophy mutations in human engineered heart muscle by single-site genome editing. *Sci. Adv.* **4**, eaap9004 (2018). 29404407 10.1126/sciadv.aap9004 PMC5796795

[123] Marini, V. et al. Long-term culture of patient-derived cardiac organoids recapitulated Duchenne muscular dystrophy cardiomyopathy and disease progression. *Front. Cell Dev. Biol.* **10**, 878311 (2022). 36035984 10.3389/fcell.2022.878311 PMC9403515

[124] Kim, C. et al. Studying arrhythmogenic right ventricular dysplasia with patient-specific iPSCs. *Nature* **494**, 105–110 (2013). 23354045 10.1038/nature11799 PMC3753229

[125] Blazeski, A. et al. Engineered heart slice model of arrhythmogenic cardiomyopathy using plakophilin-2 mutant myocytes. *Tissue Eng. Part A* **25**, 725–735 (2019). 30520705 10.1089/ten.tea.2018.0272 PMC6535962

[126] Bliley, J. M. et al. Dynamic loading of human engineered heart tissue enhances contractile function and drives a desmosome-linked disease phenotype. *Sci. Transl. Med.* **13**, eabd1817 (2021). 34290054 10.1126/scitranslmed.abd1817

[127] Wang, B. Z. et al. Engineered cardiac tissue model of restrictive cardiomyopathy for drug discovery. *Cell Rep. Med.* **4**, 100976 (2023). 10.1016/j.xcrm.2023.100976 PMC10040415 36921598

[128] Feng, W. et al. Computational profiling of hiPSC-derived heart organoids reveals chamber defects associated with NKX2-5 deficiency. *Commun. Biol.* **5**, 399 (2022). 35488063 10.1038/s42003-022-03346-4 PMC9054831

[129] Birket, M. J. et al. A human stem cell model of Fabry disease implicates LIMP-2 accumulation in cardiomyocyte pathology. *Stem Cell Rep.* **13**, 380–393 (2019). 10.1016/j.stemcr.2019.07.004 PMC6700557 31378672

[130] Hashem, S. I. et al. Brief report: Oxidative stress mediates cardiomyocyte apoptosis in a human model of Danon disease and heart failure. *Stem Cells* **33**, 2343–2350 (2015). 25826782 10.1002/stem.2015 PMC4651661

[131] Barndt, R. J. et al. Metabolic maturation exaggerates abnormal calcium handling in a Lamp2 knockout human pluripotent stem cell-derived cardiomyocyte model of Danon Disease. *Biomolecules* **13**, 69 (2022). 36671453 10.3390/biom13010069 PMC9855424

[132] Raval, K. K. et al. Pompe disease results in a Golgi-based glycosylation deficit in human induced pluripotent stem cell-derived cardiomyocytes. *J. Biol. Chem.* **290**, 3121–3136 (2015). 25488666 10.1074/jbc.M114.628628 PMC4317045

[133] Richards, D. J. et al. Human cardiac organoids for the modelling of myocardial infarction and drug cardiotoxicity. *Nat. Biomed. Eng.* **4**, 446–462 (2020). 32284552 10.1038/s41551-020-0539-4 PMC7422941

[134] Song, M. et al. Modeling acute myocardial infarction and cardiac fibrosis using human induced pluripotent stem cell-derived multi-cellular heart organoids. *Cell Death Dis.* **15**, 308 (2024). 38693114 10.1038/s41419-024-06703-9 PMC11063052

[135] Sebastião, M. J. et al. Bioreactor-based 3D human myocardial ischemia/reperfusion in vitro model: a novel tool to unveil key paracrine factors upon acute myocardial infarction. *Transl. Res.* **215**, 57–74 (2020). 31541616 10.1016/j.trsl.2019.09.001

[136] Veldhuizen, J. et al. Cardiac ischemia on-a-chip to investigate cellular and molecular response of myocardial tissue under hypoxia. *Biomaterials* **281**, 121336 (2022). 35026670 10.1016/j.biomaterials.2021.121336 PMC10440189

[137] Tiburcy, M. et al. Defined engineered human myocardium with advanced maturation for applications in heart failure modeling and repair. *Circulation* **135**, 1832–1847 (2017). 28167635 10.1161/CIRCULATIONAHA.116.024145 PMC5501412

[138] Lemme, M. et al. Chronic intermittent tachypacing by an optogenetic approach induces arrhythmia vulnerability in human engineered heart tissue. *Cardiovasc. Res.* **116**, 1487–1499 (2020). 31598634 10.1093/cvr/cvz245 PMC7314638

[139] Drawnel, F. M. et al. Disease modeling and phenotypic drug screening for diabetic cardiomyopathy using human induced pluripotent stem cells. *Cell Rep.* **9**, 810–820 (2014). 25437537 10.1016/j.celrep.2014.09.055

[140] Patel, C. N. et al. Strategies for redesigning withdrawn drugs to enhance therapeutic efficacy and safety: a review. *Wiley Interdiscip. Rev. Comput. Mol. Sci.* **15**, e70004 (2025).

[141] Di Cio, S., Marhuenda, E., Haddrick, M. & Gautrot, J. E. Vascularised cardiac spheroids-on-a-chip for testing the toxicity of therapeutics. *Sci. Rep.* **14**, 3370 (2024). 38336810 10.1038/s41598-024-53678-w PMC10858047

[142] Mozneb, M. et al. Multi-lineage heart-chip models drug cardiotoxicity and enhances maturation of human stem cell-derived cardiovascular cells. *Lab Chip* **24**, 869–881 (2024). 38252454 10.1039/d3lc00745f PMC12015978

[143] Pabel, S. et al. Long-term effects of empagliflozin on excitation-contraction-coupling in human induced pluripotent stem cell cardiomyocytes. *J. Mol. Med.* **98**, 1689–1700 (2020). 33034709 10.1007/s00109-020-01989-6 PMC7679329

[144] Hashimoto, H., Olson, E. N. & Bassel-Duby, R. Therapeutic approaches for cardiac regeneration and repair. *Nat. Rev. Cardiol.* **15**, 585–600 (2018). 29872165 10.1038/s41569-018-0036-6 PMC6241533

[145] Coulombe, K. L., Bajpai, V. K., Andreadis, S. T. & Murry, C. E. Heart regeneration with engineered myocardial tissue. *Annu. Rev. Biomed. Eng.* **16**, 1–28 (2014). 24819474 10.1146/annurev-bioeng-071812-152344 PMC4213953

[146] Chien, K. R. et al. Regenerating the field of cardiovascular cell therapy. *Nat. Biotechnol.* **37**, 232–237 (2019). 30778231 10.1038/s41587-019-0042-1

[147] Riegler, J., Gillich, A., Shen, Q., Gold, J. D. & Wu, J. C. Cardiac tissue slice transplantation as a model to assess tissue-engineered graft thickness, survival, and function. *Circulation* **130**, S77–S86 (2014). 25200059 10.1161/CIRCULATIONAHA.113.007920 PMC4353847

[148] Riegler, J. et al. Human engineered heart muscles engraft and survive long term in a rodent myocardial infarction model. *Circ. Res.* **117**, 720–730 (2015). 26291556 10.1161/CIRCRESAHA.115.306985 PMC4679370

[149] Huethorst, E. et al. Evidence for intermittent coupling of intramyocardial small, engineered heart tissues acutely implanted into rabbit myocardium. *Cardiovasc. Res.* **121**, 1697–1711 (2025). 10.1093/cvr/cvaf034 PMC12477673 40036828

[150] Silver, S. E. et al. Hypoimmunogenic hPSC-derived cardiac organoids for immune evasion and heart repair. Preprint at *bioRxiv*10.1101/2025.04.09.648007 (2025).

[151] Gao, L. et al. Myocardial tissue engineering with cells derived from human-induced pluripotent stem cells and a native-like, high-resolution, 3-dimensionally printed scaffold. *Circ. Res.* **120**, 1318–1325 (2017). 28069694 10.1161/CIRCRESAHA.116.310277 PMC5392171

[152] Zhang, D. et al. Tissue-engineered cardiac patch for advanced functional maturation of human ESC-derived cardiomyocytes. *Biomaterials* **34**, 5813–5820 (2013). 23642535 10.1016/j.biomaterials.2013.04.026 PMC3660435

[153] Han, X. et al. Generation of hypoimmunogenic human pluripotent stem cells. *Proc. Natl. Acad. Sci. USA* **116**, 10441–10446 (2019). 31040209 10.1073/pnas.1902566116 PMC6535035

[154] Tsuneyoshi, N. et al. Hypoimmunogenic human iPSCs expressing HLA-G, PD-L1, and PD-L2 evade innate and adaptive immunity. *Stem Cell Res. Ther.* **15**, 193 (2024). 38956724 10.1186/s13287-024-03810-4 PMC11218117

[155] Tereshchenko, Y., Petkov, S. G. & Behr, R. The efficiency of in vitro differentiation of primate iPSCs into cardiomyocytes depending on their cell seeding density and cell line specificity. *Int. J. Mol. Sci.* **25**, 8449 (2024). 39126016 10.3390/ijms25158449 PMC11312487

[156] Ismaili, D. et al. Human induced pluripotent stem cell-derived cardiomyocytes as an electrophysiological model: Opportunities and challenges—the Hamburg perspective. *Front. Physiol.* **14**, 1132165 (2023). 36875015 10.3389/fphys.2023.1132165 PMC9978010

[157] Grebenyuk, S. et al. Large-scale perfused tissues via synthetic 3D soft microfluidics. *Nat. Commun.* **14**, 193 (2023). 36635264 10.1038/s41467-022-35619-1 PMC9837048

[158] Juhola, M., Penttinen, K., Joutsijoki, H. & Aalto-Setälä, K. Analysis of drug effects on iPSC cardiomyocytes with machine learning. *Ann. Biomed. Eng.* **49**, 129–138 (2021). 32367466 10.1007/s10439-020-02521-0 PMC7773623

[159] Yang, H., Yang, Y., Kiskin, F. N., Shen, M. & Zhang, J. Z. Recent advances in regulating the proliferation or maturation of human-induced pluripotent stem cell-derived cardiomyocytes. *Stem Cell Res. Ther.* **14**, 228 (2023). 37649113 10.1186/s13287-023-03470-w PMC10469435

[160] Konala, V. B. R. et al. CiPA-qualified human iPSC-derived cardiomyocytes: A new frontier in toxicity testing by evaluating drug-induced arrhythmias. *Toxicol. In Vitro* **108**, 106100 (2025). 10.1016/j.tiv.2025.106100 40482844

[161] Kowalczewski, A. et al. Integrating nonlinear analysis and machine learning for human induced pluripotent stem cell-based drug cardiotoxicity testing. *J. Tissue Eng. Regen. Med.* **16**, 732–743 (2022). 35621199 10.1002/term.3325 PMC9719611

[162] Gerdes, A. M. et al. Structural remodeling of cardiac myocytes in patients with ischemic cardiomyopathy. *Circulation* **86**, 426–430 (1992). 1638711 10.1161/01.cir.86.2.426

[163] Ball, A. J. & Levine, F. Telomere-independent cellular senescence in human fetal cardiomyocytes. *Aging Cell* **4**, 21–30 (2005). 15659210 10.1111/j.1474-9728.2004.00137.x

[164] Shih, H.-T. Anatomy of the action potential in the heart. *Tex. Heart Inst. J.* **21**, 30 (1994). 7514060 PMC325129

[165] Vigmond, E. J., Massé, S., Roney, C. H., Bayer, J. D. & Nanthakumar, K. The accuracy of cardiac surface conduction velocity measurements. *Clin. Electrophysiol.* **11**, 694–705 (2025). 10.1016/j.jacep.2024.11.004 39818672

[166] Lu, X. et al. Measuring local gradients of intramitochondrial [Ca2+] in cardiac myocytes during sarcoplasmic reticulum Ca2+ release. *Circ. Res.* **112**, 424–431 (2013). 23243207 10.1161/CIRCRESAHA.111.300501 PMC3566246

[167] Pioner, J. M. et al. Optical investigation of action potential and calcium handling maturation of hiPSC-cardiomyocytes on biomimetic substrates. *Int. J. Mol. Sci.* **20**, 3799 (2019). 31382622 10.3390/ijms20153799 PMC6695920

[168] Hwang, H. S. et al. Comparable calcium handling of human iPSC-derived cardiomyocytes generated by multiple laboratories. *J. Mol. Cell. Cardiol.* **85**, 79–88 (2015). 25982839 10.1016/j.yjmcc.2015.05.003 PMC4530041

[169] Alvarez, J. A. E., Jafri, M. S. & Ullah, A. Local control model of a human ventricular myocyte: an exploration of frequency-dependent changes and calcium sparks. *Biomolecules* **13**, 1259 (2023). 37627324 10.3390/biom13081259 PMC10452762

[170] Hasenfuss, G. et al. Energetics of isometric force development in control and volume-overload human myocardium. Comparison with animal species. *Circ. Res.* **68**, 836–846 (1991). 1742869 10.1161/01.res.68.3.836

[171] King, J. & Lowery, D. R. *Physiology, Cardiac Output* (StatPearls, 2017).

[172] Lopaschuk, G. D., Spafford, M. A. & Marsh, D. R. Glycolysis is predominant source of myocardial ATP production immediately after birth. *Am. J. Physiol. Heart Circ. Physiol.* **261**, H1698–H1705 (1991). 10.1152/ajpheart.1991.261.6.H1698 1750528

[173] Scuderi, G. J. & Butcher, J. Naturally engineered maturation of cardiomyocytes. *Front. Cell Dev. Biol.* **5**, 50 (2017). 28529939 10.3389/fcell.2017.00050 PMC5418234

[174] Jacot, J. G., McCulloch, A. D. & Omens, J. H. Substrate stiffness affects the functional maturation of neonatal rat ventricular myocytes. *Biophys. J.* **95**, 3479–3487 (2008). 18586852 10.1529/biophysj.107.124545 PMC2547444

[175] Bhana, B. et al. Influence of substrate stiffness on the phenotype of heart cells. *Biotechnol. Bioeng.* **105**, 1148–1160 (2010). 20014437 10.1002/bit.22647

[176] Kijlstra, J. D. et al. Integrated analysis of contractile kinetics, force generation, and electrical activity in single human stem cell-derived cardiomyocytes. *Stem Cell Rep.* **5**, 1226–1238 (2015). 10.1016/j.stemcr.2015.10.017 PMC4682285 26626178

[177] Yang, X. et al. Tri-iodo-l-thyronine promotes the maturation of human cardiomyocytes-derived from induced pluripotent stem cells. *J. Mol. Cell. Cardiol.* **72**, 296–304 (2014). 24735830 10.1016/j.yjmcc.2014.04.005 PMC4041732

[178] Feyen, D. A. et al. Metabolic maturation media improve physiological function of human iPSC-derived cardiomyocytes. *Cell Rep.* **32**, 107925 (2020). 10.1016/j.celrep.2020.107925 PMC7437654 32697997

[179] Yadid, M. et al. Endothelial extracellular vesicles contain protective proteins and rescue ischemia-reperfusion injury in a human heart-on-chip. *Sci. Transl. Med.* **12**, eaax8005 (2020). 33055246 10.1126/scitranslmed.aax8005 PMC8969368

[180] Kim, D.-S. et al. Highly durable crack sensor integrated with silicone rubber cantilever for measuring cardiac contractility. *Nat. Commun.* **11**, 535 (2020). 31988308 10.1038/s41467-019-14019-y PMC6985253

[181] Li, J. et al. Circulating re-entrant waves promote maturation of hiPSC-derived cardiomyocytes in self-organized tissue ring. *Commun. Biol.* **3**, 122 (2020). 32170165 10.1038/s42003-020-0853-0 PMC7070090

[182] Goldfracht, I. et al. Generating ring-shaped engineered heart tissues from ventricular and atrial human pluripotent stem cell-derived cardiomyocytes. *Nat. Commun.* **11**, 75 (2020). 31911598 10.1038/s41467-019-13868-x PMC6946709

[183] Jackman, C. P., Carlson, A. L. & Bursac, N. Dynamic culture yields engineered myocardium with near-adult functional output. *Biomaterials* **111**, 66–79 (2016). 27723557 10.1016/j.biomaterials.2016.09.024 PMC5074846

[184] Wang, E. Y. et al. Biowire model of interstitial and focal cardiac fibrosis. *ACS Cent. Sci.* **5**, 1146–1158 (2019). 31403068 10.1021/acscentsci.9b00052 PMC6661857

[185] Ma, Z. et al. Contractile deficits in engineered cardiac microtissues as a result of MYBPC3 deficiency and mechanical overload. *Nat. Biomed. Eng.* **2**, 955–967 (2018). 31015724 10.1038/s41551-018-0280-4 PMC6482859

[186] Shadrin, I. Y. et al. Cardiopatch platform enables maturation and scale-up of human pluripotent stem cell-derived engineered heart tissues. *Nat. Commun.* **8**, 1825 (2017). 29184059 10.1038/s41467-017-01946-x PMC5705709

[187] Visone, R. et al. A microscale biomimetic platform for generation and electro-mechanical stimulation of 3D cardiac microtissues. *APL Bioeng.* **2**, 046102 (2018). 10.1063/1.5037968 PMC6481729 31069324

[188] Schneider, O. et al. Fusing spheroids to aligned μ-tissues in a heart-on-chip featuring oxygen sensing and electrical pacing capabilities. *Mater. Today Bio* **15**, 100280 (2022). 10.1016/j.mtbio.2022.100280 PMC9120495 35601892

[189] Hinson, J. T. et al. Integrative analysis of PRKAG2 cardiomyopathy iPS and microtissue models identifies AMPK as a regulator of metabolism, survival, and fibrosis. *Cell Rep.* **17**, 3292–3304 (2016). 28009297 10.1016/j.celrep.2016.11.066 PMC5193246

[190] Prondzynski, M. et al. Disease modeling of a mutation in α-actinin 2 guides clinical therapy in hypertrophic cardiomyopathy. *EMBO Mol. Med.* **11**, e11115 (2019). 31680489 10.15252/emmm.201911115 PMC6895603

[191] Rohr, S., Schölly, D. & Kleber, A. G. Patterned growth of neonatal rat heart cells in culture. Morphological and electrophysiological characterization. *Circ. Res.* **68**, 114–130 (1991). 1984856 10.1161/01.res.68.1.114

[192] Rohr, S., Kucera, J. P., Fast, V. G. & Kléber, A. G. Paradoxical improvement of impulse conduction in cardiac tissue by partial cellular uncoupling. *Science* **275**, 841–844 (1997). 9012353 10.1126/science.275.5301.841

[193] Fink, C. et al. Chronic stretch of engineered heart tissue induces hypertrophy and functional improvement. *FASEB J.* **14**, 669–679 (2000). 10744624 10.1096/fasebj.14.5.669

[194] Zimmermann, W.-H. et al. Cardiac grafting of engineered heart tissue in syngenic rats. *Circulation* **106**, I151-7 (2002). 12354725

[195] Radisic, M. et al. Functional assembly of engineered myocardium by electrical stimulation of cardiac myocytes cultured on scaffolds. *Proc. Natl. Acad. Sci. USA* **101**, 18129–18134 (2004). 15604141 10.1073/pnas.0407817101 PMC539727

[196] Zhang, J. et al. Functional cardiomyocytes derived from human induced pluripotent stem cells. *Circ. Res.* **104**, e30–e41 (2009). 19213953 10.1161/CIRCRESAHA.108.192237 PMC2741334

